# ULI-ssDRIP-seq revealed R-loop dynamics during vertebrate early embryogenesis

**DOI:** 10.1016/j.cellin.2024.100179

**Published:** 2024-06-01

**Authors:** Wei Xu, Xin Liu, Jinjin Li, Changbin Sun, Luxi Chen, Jincong Zhou, Kuan Li, Qin Li, Anming Meng, Qianwen Sun

**Affiliations:** a School of Life Sciences, Tsinghua University, Beijing, 100084, China; b Shenzhen Branch, Guangdong Laboratory of Lingnan Modern Agriculture, Genome Analysis Laboratory of the Ministry of Agriculture and Rural Affairs, Agricultural Genomics Institute at Shenzhen, Chinese Academy of Agricultural Sciences, Shenzhen, 518120, China; c Tsinghua-Peking Center for Life Sciences, Beijing, 100084, China

**Keywords:** R-loop, Zygotic genome activation, Embryonic development, Next generation sequencing, Embryogenesis

## Abstract

R-loop, a chromatin structure containing one RNA:DNA hybrid and one unpaired single-stranded DNA, plays multiple biological roles. However, due to technical limitations, the landscapes and potential functions of R-loops during embryogenesis remain elusive. Here, we developed a quantitative and high-resolution ultra-low input R-loop profiling method, named ULI-ssDRIP-seq, which can map global R-loops with as few as 1000 cells. By using ULI-ssDRIP-seq, we reveal the R-loop dynamics in the zebrafish from gametes to early embryos. In oocytes, the R-loop level is relatively low in most regions of the nuclear genome, except maternal-inherited rDNA and mitochondrial genome. The correlation between R-loop and CG methylation dynamics during early development is relatively weak. Furthermore, either up- or down-regulation of global R-loops by knockdown or overexpression of RNase H1 causes a delay of embryonic development with dramatic expression changes in zygotic and maternal genes. This study provides comprehensive R-loop landscapes during early vertebrate embryogenesis and demonstrates the implication of R-loops in embryonic development.

## Highlights

• Develop a quantitative, extra-high-efficiency and near-single-nucleotide-resolution R-loop profiling method, ULI-ssDRIP-seq.
• Construct the first R-loop atlas of parental-to-zygotic transition in vertebrate.
• Reveal the roles of balanced R-loop dynamics in ensuring early embryonic development.

## Graphical abstract

**Figure:**
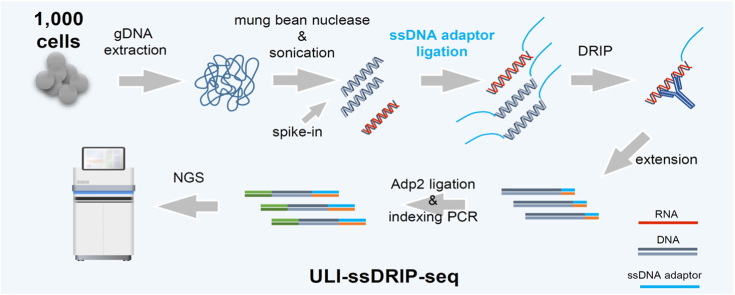

## 1 Introduction

R-loop is a pervasive genome structure containing one RNA:DNA hybrid and the other unpaired single-stranded DNA (ssDNA) (Chedin, 2016; Garcia-Muse & Aguilera, 2019; Niehrs & Luke, 2020; Petermann et al., 2022; Santos-Pereira & Aguilera, 2015; Zhou, Zhang, & Sun, 2022). R-loops play important roles in many biological processes, including RNA transcription, epigenetic modifications, DNA repair, and genome integrity (Brickner et al., 2022; Chedin, 2016; Crossley et al., 2019; Garcia-Muse & Aguilera, 2019; Li et al., 2023; Niehrs & Luke, 2020; Petermann et al., 2022; Santos-Pereira & Aguilera, 2015; Zhou et al., 2023). The essential functions of R-loops in regulating development have been revealed. In plants, R-loops are important for flower, pollen, chloroplast and root development (Cheng et al., 2021; Li et al., 2023; Shafiq et al., 2017; Sun et al., 2013; Wang, Li, et al., 2021; Yang et al., 2017, 2020; Zhang et al., 2024; Zhou et al., 2023). In animals, R-loops are also involved in the differentiation of embryonic stem cells (ESCs) (Chen et al., 2015) and breast luminal epithelial cells (Chiang et al., 2019; Zhang et al., 2017), and reprogramming somatic cells to induced pluripotent stem cells (iPSCs) (Li et al., 2020). In zebrafish (*Danio rerio*), R-loops can regulate early embryonic development by orchestrating tRNA gene transcription(Chen et al., 2021), and are also involved in the developmental regulation of embryonic neurons (Sorrells et al., 2018), hematopoietic stem and progenitor cells (HSPCs) (Weinreb et al., 2021).

Embryogenesis starts from fertilization when male and female gametes meet, giving a new life. Materials from the mother in animals usually support embryonic development through the first several cell divisions. Parental to zygotic transition is the first crucial event for early development, which is primarily defined by the onset of both maternal mRNA decay and zygotic genome activation (ZGA) (Vastenhouw et al., 2019). The zebrafish is a typical and well-studied model for ZGA, and the initiation of major ZGA in zebrafish takes place at approximately the 10th cell division (1000-cell stage. about 3 h postfertilization), which is quite late when compared with mouse (2-cell stage) and human (4-cell stage) embryos (Jukam et al., 2017). Thus, a relatively large number of cells before the major wave of ZGA is highly favorable for chromatin biology studies. Genomic studies have revealed similarities and differences between zebrafish and other species at this crucial time point, including the aspects of epigenetic modifications, chromatin accessibility, and chromosome 3D structure (Jambhekar et al., 2019; Schulz & Harrison, 2019). Histone modifications that mark regulatory elements of target genes, such as H3K27ac, H3K4me3, and H3K27me3, undergo widespread reprogramming on enhancer and promoter regions during the parental-to-zygotic transition (Zhang et al., 2018). Furthermore, unlike in mice and humans, whose genomes undergo drastic global DNA demethylation before ZGA, the zebrafish genome remains highly methylated, and the maternal oocyte methylome is converted to a pattern highly resembling that of sperm after fertilization (Jiang et al., 2013; Potok et al., 2013; Wu et al., 2021). Although ZGA in early zebrafish embryos has been elucidated from a variety of aspects, the underlying mechanisms remain elusive. As R-loop is correlated with plenty of genome regulation events (Al-Hadid & Yang, 2016; Crossley et al., 2019; Garcia-Muse & Aguilera, 2019), we speculate that it might play potential roles in early embryogenesis.

In the last decade, many methods have been developed to profile global R-loops (Crossley et al., 2019; Zhou, Wang, & Sun, 2022), such as DRIP-seq (Ginno et al., 2012), S1-DRIP-seq (Wahba et al., 2016), DRIPc-seq (Sanz et al., 2016), ssDRIP-seq (Xu et al., 2017), R-ChIP (Chen et al., 2017), bisDRIP-seq (Dumelie & Jaffrey, 2017), qDRIP-seq (Crossley et al., 2020), MapR (Yan & Sarma, 2020), and R loop CUT&Tag (Wang et al., 2021). Among these, S9.6 monoclonal antibody has been widely used for immunoprecipitation of RNA:DNA hybrids, as it recognizes the minor groove of RNA:DNA hybrid and prefers the DNA in the RNA:DNA hybrid (Bou-Nader et al., 2022; Li et al., 2022). Although different R-loop profiling methods have their own advantages and disadvantages (Crossley et al., 2019; Zhou et al., 2022), all of them have limitations in their application to ultra-low-input samples, such as early embryos. To investigate the dynamics and potential functions of R-loop during early development, we developed an extra-high-efficiency and near-single-nucleotide-resolution R-loop profiling method, ULI-ssDRIP-seq (Ultra-Low-Input single-strand DNA ligation-based library construction from DNA:RNA hybrid ImmunoPrecipitation, followed by sequencing).

## 2 Results

### 2.1 Mung bean nuclease digestion boosts the resolution of genome-wide R-loop profiling

**Fig. 1:**
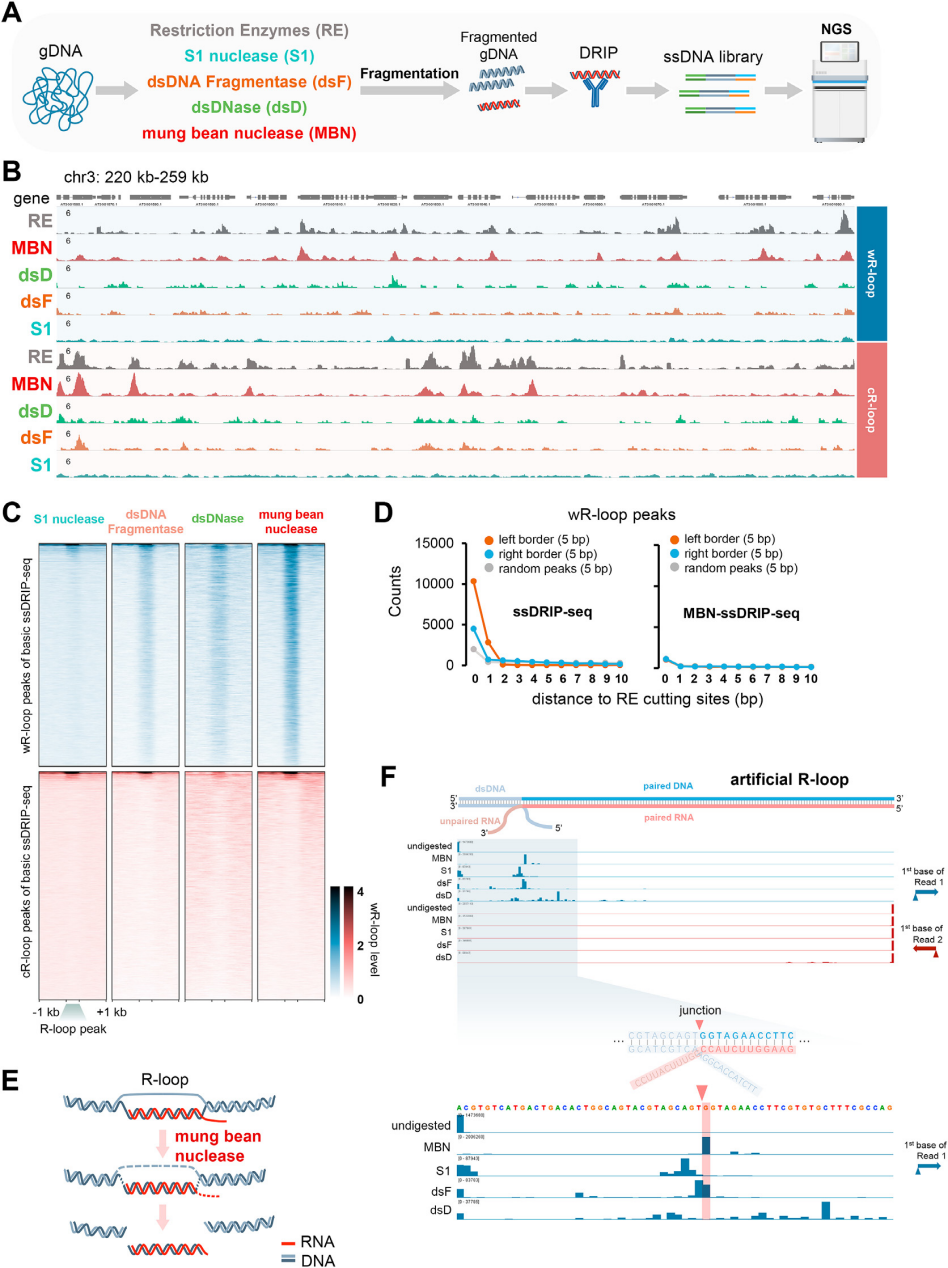
**Improvements ofssDRIP-seqby optimizing the genomic DNA fragmentation.** **A.** Schematic of the exploration experiment for the improvement of ssDRIP-seq. Briefly, the gDNA digestion step was first performed by using S1 nuclease, dsDNA Fragmentase, dsDNase, mung bean nuclease, and restriction enzymes. Then gDNA was fragmented by sonication, followed by DRIP, ssDNA library preparation, and sequencing. **B.** IGV snapshot of Arabidopsis wR-loop (Watson R-loop, with a Watson strand DNA as the unpaired ssDNA) and cR-loop (Crick R-loop, with a Crick strand DNA as the unpaired ssDNA) signals from basic ssDRIP-seq using restriction enzymes (RE, grey) and other modified ssDRIP-seq using mung bean nuclease (MBN, red), dsDNase (dsD, light green), dsDNA Fragmentase (dsF, brown), or S1 nuclease (S1, cyan) for gDNA treatment. **C.** Heatmaps of Arabidopsis wR-loop signals of modified ssDRIP-seq using S1 nuclease, dsDNA Fragmentase, dsDNase, or mung bean nuclease. The signals around wR-loop (upper, blue) or cR-loop (lower, red) peaks of basic ssDRIP-seq were shown. **D.** Statistics of boundaries of wR-loop peaks (extended ± 5 bp) from basic ssDRIP-seq (left) or MBN-ssDRIP-seq (right, modified ssDRIP-seq using mung bean nuclease) with different distances from restriction enzyme cutting sites (*Dde*I, *Mse*I, *Nla*III and *Mbo*I). 0 bp means overlapping. **E.** Supposed model of R-loop treatment by mung bean nuclease. **F.** MBN-ssDRIP-seq result of artificial half R-loop (seen in Fig. S1H). The artificial R-loop was first treated with mung bean nuclease (MBN), S1 nuclease(S1), dsDNA Fragmentase (dsF), or dsDNase (dsD), followed by DRIP and ssDNA library preparation. IGV snapshot showed the counts of the first bases of Watson (blue) and Crick (red) reads. The junction and expected boundaries was marked by red triangle and red shadow respectively.

ssDRIP-seq has been successfully used to profile R-loop landscapes in various organisms (Chen et al., 2021; Li et al., 2020; Liu et al., 2021; Liu & Sun, 2021; Munden et al., 2022; Xiao et al., 2022; Xu et al., 2017, 2020; Yan et al., 2020; Yang et al., 2019; Yuan et al., 2019). However, some shortcomings limit the usage of ssDRIP-seq, for example, the resolution is not at the single-nucleotide level and there is a restriction on minimum input (normally 2 × 10^5^–1 × 10^7^ animal cells (Chen et al., 2021; Li et al., 2020; Yang et al., 2019)). The resolution of ssDRIP-seq is mainly limited by the fragmentation process that is based on the combination of high-frequency restriction enzymes, in which the cleavage sites determine the boundaries of R-loops (Fig. S1A). To address this issue, several nucleases, including S1 nuclease, dsDNA Fragmentase, dsDNase, and mung bean nuclease were tested individually to replace the group of restriction enzymes (Fig. 1A, see Methods). As S1 nuclease and mung bean nuclease digest ssDNA and/or ssRNA only, they were assumed to be able to remove the ssDNA part and cut the junctions between RNA:DNA hybrid and dsDNA (double-stranded DNA) of the existing R-loop structure (Fig. S1A). While dsDNA Fragmentase and dsDNase digested dsDNA only, we expected that one of them could remove the dsDNA part from an R-loop structure and leave a ssDNA and an RNA:DNA hybrid strand (Fig. S1A). After digestion with each of these enzymes respectively, the remaining steps were performed following ssDRIP-seq as described previously (Xu et al., 2017).

After sequencing and data analysis, snapshots of basic [represented by RE (restriction enzymes)] and modified ssDRIP-seq signals [represented by MBN (mung bean nuclease), dsD (dsDNase), dsF (dsDNA Fragmentase), and S1 (S1 nuclease), respectively] are shown in Fig. 1B and Fig. S1B. From the results, we found that the genomic distribution of R-loop signals from MBN-ssDRIP-seq (modified ssDRIP-seq using mung bean nuclease) was very similar to that from ssDRIP-seq, while signals of the other modified ssDRIP-seq showed much less similarity (as shown in Fig. 1B and Fig. S1B). Correspondingly, the MBN-ssDRIP-seq results showed outstanding strand-specificity and the highest genome-wide enrichment of signals on R-loop peaks profiled by ssDRIP-seq (Fig. 1C). The results of correlation analysis of R-loop signal also showed that the signals of MBN-ssDRIP-seq and ssDRIP-seq were most similar (Fig. S1C). In addition, as shown by the statistical results, the peak sizes of MBN-ssDRIP-seq were closest to those of ssDRIP-seq (Fig. S1D). Furthermore, snapshots of MBN-ssDRIP-seq showed a smoother boundary of the signal than that of ssDRIP-seq (Fig. 1B and Fig. S1B). As mentioned above, the boundaries of most R-loop peaks profiled by ssDRIP-seq were located on the cleavage sites of the combined restriction enzymes, while this fragmentation bias was highly reduced by MBN-ssDRIP-seq (Fig. 1D and S1E). The meta-analysis results showed gentler boundaries of MBN-ssDRIP-seq signal compared with those of ssDRIP-seq (Fig. S1F). We then compared the sizes of each pair of R-loop peaks from MBN-ssDRIP-seq and ssDRIP-seq that overlapped with each other, and found that most of the MBN-ssDRIP-seq peaks were shorter than the corresponding ssDRIP-seq peaks (Fig. S1G).

Overall, MBN-based ssDRIP-seq could provide higher resolution R-loop signals. We then wondered whether the resolution of MBN-ssDRIP-seq could be pushed to the single-nucleotide level, as the mung bean nuclease was expected to remove the RNA and ssDNA, and cleave the junctions between RNA:DNA hybrid and dsDNA (Fig. 1E). To test this hypothesis, we designed an artificial half R-loop substrate containing a junction between hybrid and dsDNA segments for MBN-ssDRIP-seq (Fig. S1H). Sequencing results showed that the ssDNA portion was completely removed by mung bean nuclease, thus separating relatively intact RNA:DNA hybrids from R-loops (Fig. 1F). In contrast, the boundaries generated by digestion with S1 nuclease, dsDNA Fragmentase or dsDNase showed varying degrees of deviation (Fig. 1F). These results can fully explain the above sequencing results shown in Fig. 1B and C that pretreatment with mung bean nuclease can dramatically improve the performance of ssDRIP-seq and achieve much higher resolution to single-nucleotide level (Fig. 1E and F).

### 2.2 Establishment of the ultra-low-input R-loop profiling Method

**Fig. 2:**
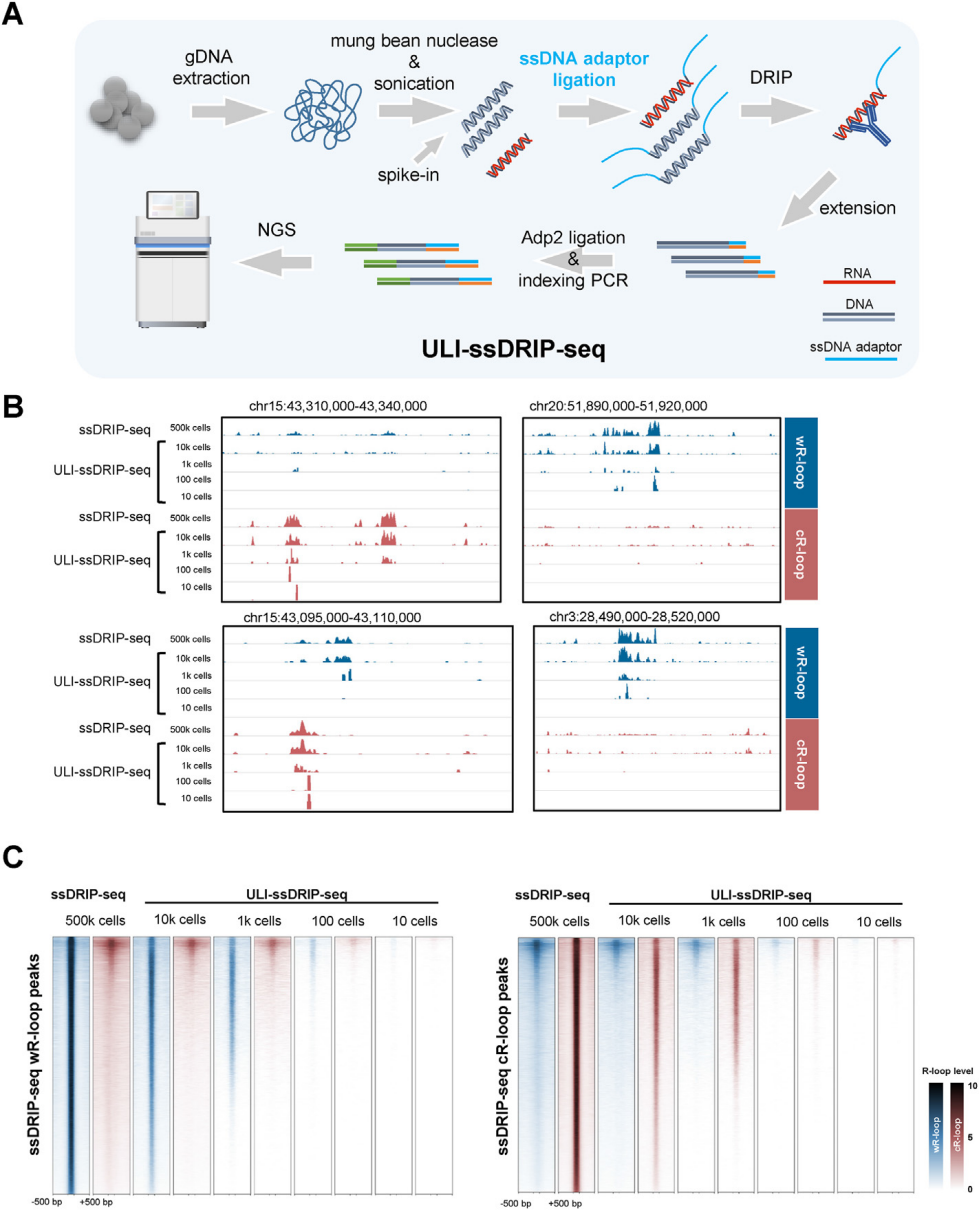
**Establishment ofULI-ssDRIP-seq,the R-loop profiling method forUltra-low-inputsamples.** **A.** Schematic of workflow of ULI-ssDRIP-seq method. **B.** IGV snapshots of zebrafish wR-loop (blue) and cR-loop (red) signals from ssDRIP-seq and ULI-ssDRIP-seq with different quantities of input. **C.** Metaplots and heatmaps of zebrafish wR-loop (blue) and cR-loop (red) signals from ssDRIP-seq (500k cells) and ULI-ssDRIP-seq with different quantities of input (10k, 1k, 100, or 10 cells). The signals around wR-loop (left) and cR-loop (right) peaks of 500k cells ssDRIP-seq were shown.

Reported genome-wide R-loop profiling methods require at least 50,000 cells (Chen et al., 2017; Crossley et al., 2020; Dumelie & Jaffrey, 2017; Ginno et al., 2012; Sanz et al., 2016; Wahba et al., 2016; Wang et al., 2021; Wu et al., 2022; Xu et al., 2017; Yan & Sarma, 2020), which limits their application for studying R-loops in samples with a limited number of cells. During early embryonic development, cells are very precious. To understand the dynamics of R-loops during parental-to-zygotic transition, we combined mung bean nuclease digestion and reconstructed the frame of ssDRIP-seq for ultra-low-input samples (ULI-ssDRIP-seq shown in Fig. 2A). To avoid losing DNA samples during DNA extraction and transfer between tubes, fragmented gDNA was ligated with the first adaptor and then directly immunoprecipitated with the R-loop antibody S9.6, followed by an extension step on beads without precipitation to purify immunoprecipitated DNA (Fig. 2A). Compared to ssDRIP-seq, ULI-ssDRIP-seq only required one precipitation step (after mung bean nuclease digestion), which can minimize sample loss.

To test the performance of this optimization, we used different amounts of genomic DNA (gDNA, from 37 pg to 37 ng) from zebrafish muscle tissue, simulating the gDNA from 10 to 10,000 somatic cells, as the starting input for ULI-ssDRIP-seq. The R-loop profiling of gDNA from ∼500,000 muscle cells was performed by basic ssDRIP-seq as the positive control. The sequencing results, presented by Pearson's correlation coefficient, showed high positive correlations between ssDRIP-seq and ULI-ssDRIP-seq, while a low correlation was found between the negative control RNase H-treated samples and others (Fig. S2A). The R-loop signals from ssDRIP-seq (500,000 cells, 500k cells) and ULI-ssDRIP-seq (10,000 cells, 10k cells) were very similar (Fig. 2B and Fig. S2B), indicating the high performance and reliability of ULI-ssDRIP-seq for R-loop profiling. Although the R-loop signals in the 1k cell group were not exactly the same as those in the 10k cell group, they showed good reproducibility (Fig. 2B and Fig. S2B). Importantly, sparser R-loop signals were also found in the 10 and 100 cell groups, with a distribution consistent with that of ssDRIP-seq (Fig. 2B and Fig. S2B).

For the reliability and reproducibility of ULI-ssDRIP-seq, the meta-analysis clearly showed that the R-loop signals from ssDRIP-seq or 10k cell group from ULI-ssDRIP-seq were highly enriched in all R-loop peaks from ssDRIP-seq, while signals from 1k, 100, and 10 cell groups were enriched in ∼70%, ∼40%, and ∼10% of R-loop peaks, respectively (Fig. 2C). All groups exhibited significant differences between wR-loop (Watson R-loop, with a Watson strand DNA as the unpaired ssDNA) and cR-loop (Crick R-loop, with a Crick strand DNA as the unpaired ssDNA) (Fig. 2C), suggesting that ULI-ssDRIP-seq could detect R-loops in a strand-specific manner, the same with ssDRIP-seq. We supposed that the lower signal level in the 1k, 100, and 10 cell groups might be due to the lower amounts of R-loops in the tested ultra-low-input samples, rather than the lower adaptability for ultra-low-input samples. To prove this, we further counted the reads mapped in, or out of R-loop peaks. The statistical results showed that 47–60% of reads from ssDRIP-seq or ULI-ssDRIP-seq groups were located in R-loop peaks, especially the 1k and 100 cell groups, which achieved almost the same mapping rate as that of ssDRIP-seq control (Fig. S2C). We applied principal component analysis (PCA) and found that RNase H treatment group diverged from the untreated groups, which demonstrated that the ULI-ssDRIP-seq signals of low-input samples were erased by RNase H (Fig. S2D).

Taken together, these results suggest that ULI-ssDRIP-seq is stable and reliable for R-loop profiling in very few cells, and suitable for studying the R-loop dynamics and functions during early embryonic development.

### 2.3 Global R-loop landscapes in zebrafish somatic cells and early embryos

**Fig. 3:**
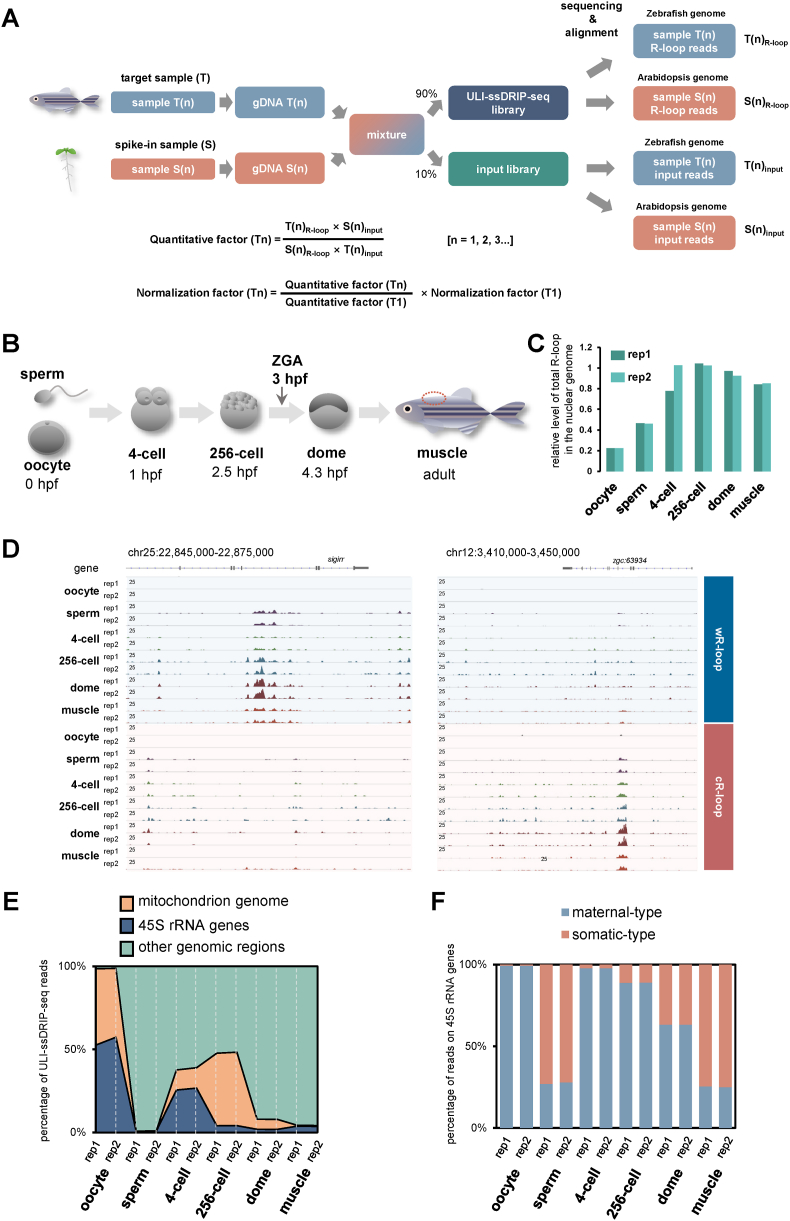
**R-loop landscapes duringparental-to-zygotictransition.** **A.** Schematic of the quantification method used in ULI-ssDRIP-seq. **B.** Schematic of early developmental stages examined in this study, including sperm, oocyte, 4-cell, 256-cell, dome stages and dorsal muscle (marked with red circle) from adult fish. Hours post fertilization, hpf. **C.** Relative levels of total R-loops in the nuclear genome from two biological replications of oocyte, sperm, 4-cell, 256-cell, dome, and muscle groups respectively. **D.** IGV snapshots of wR-loop and cR-loop signals from oocytes, sperms, 4-cell, 256-cell, dome embryos, and muscles (T1). **E.** Percentages of ULI-ssDRIP-seq reads from two biological replications of oocyte, sperm, 4-cell, 256-cell, dome, and muscle groups, mapped to the mitochondrial, 45S rRNA genes, or other genomic regions. **F.** Percentages of ULI-ssDRIP-seq reads from two biological replications of oocyte, sperm, 4-cell, 256-cell, dome, and muscle groups, mapped to maternal-type or somatic-type 45S rRNA genes.

To quantitatively profile R-loops during early embryonic development, we further updated ULI-ssDRIP-seq by introducing the spike-in sample (Fig. 3A). Briefly, Arabidopsis seedlings were selected as the spike-in sample (S), while zebrafish samples were represented as the target sample (T). Samples T and S were mixed together and then divided into two parts, 90% for ULI-ssDRIP-seq (named ULI-ssDRIP-seq library), and the other 10% for genomic DNA sequencing library (named input library) used as background. By counting reads from ULI-ssDRIP-seq or input library mapped to the target or spike-in genome, quantitative factors were calculated and then used to correct the levels of R-loop signals among all target samples (Fig. 3A).

Using this quantitative ULI-ssDRIP-seq, R-loops were quantitatively profiled from six groups with two replicates each, including sperms, oocytes, muscles, and embryos at 4-cell stage (1 hpf), 256-cell stage (2.5 hpf), and dome stage (3.7 hpf) (Fig. 3B). First, measurement of total R-loop levels from different groups showed that, the lowest total R-loop level was in oocytes, while it was two times higher in sperms (Fig. 3C); at the 4-cell stage, the R-loop level was increased to approximately 4 times compared to that of the oocyte group, and reached a maximum at the 256-cell stage (Fig. 3C); and then, the R-loop level decreased slightly in dome and muscle groups (Fig. 3C). Principal component analysis (PCA) result showed an excellent consistency between two biological replicates from each group, while significant differences existed among groups (Fig. S3A). From the PCA results, we found that the R-loop pattern at the dome stage was dramatically different from that at the 256-cell stage (Fig. S3A). Furthermore, we found that the RNA expression level of *rnaseh1* gene, an important gene encoding the endonuclease to specifically degrade the RNA moiety in RNA:DNA hybrids (Cerritelli & Crouch, 2009), was dramatically decreased at the dome stage compared with that at the 256-cell stage (Fig. S3B). These results suggest that the extensive and intensive regulation of R-loops occurs during ZGA.

Spearman's rank correlation coefficient analysis indicated a good reproduction between two replicates from each group, except for oocytes (Fig. S3C). More differences were found between the oocyte group and the other groups (Fig. S3C), indicating a unique R-loop pattern in oocytes. The distributions of R-loops from each group were shown in Fig. 3D and S3D. We found R-loop levels at some loci were dynamic during early development, and the results were validated by DRIP-qPCR (Fig. S3D). Together, based on ULI-ssDRIP-seq, we have built an R-loop atlas during zebrafish early development.

### 2.4 R-loop distribution features during early development

As shown in Fig. 3D, the R-loop signal was extremely low in the oocyte, which seemed to contradict the quantitative result of total R-loop levels shown in Fig. 3C. We therefore further analyzed the distribution of the reads in different genomic regions. Approximately half of the ULI-ssDRIP-seq reads of oocyte group were mapped to the mitochondrial genome and the other half were mapped to 45S rRNA genes, while only a few (< 2%) of them were aligned to other regions of the nuclear genome (Fig. 3E). This result explained the extra-low R-loop levels at most genomic regions in oocyte group, as shown in Fig. 3D. However, during embryonic development, the R-loop reads mapped to 45S rDNA were decreased gradually (Fig. 3E). By analyzing the types of rRNA genes (Locati et al., 2017), we found that only maternal-type 45S rRNA genes were enriched with R-loops in oocytes, and the proportion of R-loops on somatic-type 45S rRNA genes increased during early development (Fig. 3F). Reads mapped to the mitochondrial genome showed similar trends with 45S rDNA at all examined stages, except at the 256-cell stage (Figs. S4A and S4B). We found that the mitochondrial R-loop level increased at the 256-cell stage compared to the 4-cell stage (Fig. 3E and Figs. S4A), which illustrated an activation of R-loops in the mitochondrial genome before ZGA. Interestingly, the results showed that mitochondrial R-loops were enriched on rRNA and tRNA genes, rather than protein-coding genes (Fig. S4B). As mitochondria are maternally inherited (Giles et al., 1980), these results suggest an intriguing hypothesis that the potential regulation of R-loop dynamics in early development could be governed by the maternal side. Due to the extra low R-loop level in normal genomic regions of oocytes, the oocyte group will not be included in most of the following analyses.

To further understand the pattern of R-loop distribution in the zebrafish nuclear genome, R-loop peaks were called from each group by MACS2 (Feng et al., 2012). The peak size distributions among all groups were similar (Fig. S5A), as the means or medians were very close (Fig. S5B). This result suggested that the lengths of R-loop peaks are stable in most zebrafish samples. Next, analysis of the distribution of R-loop peaks in different genomic regions showed a similar pattern among all samples (Fig. S5C). Nearly half of the peaks were located in intergenic regions, while only a small proportion of R-loop peaks (∼6%) were enriched in promoter regions of genes (Fig. S5C). To further understand the distribution of R-loops on different types of genes, we then analyzed the R-loop signals around each type of gene. The results showed slight enrichment of R-loop signals on gene body regions of protein-coding genes, rather than promoter regions (Fig. S6A), which was consistent with the result shown in Fig. S5C. The level of R-loops on protein-coding genes was lowest in sperms, and increased gradually from the 4-cell stage to the dome stage, and was highest in somatic (muscle) cells (Fig. S6A). Furthermore, analysis of other types of genes showed that a few R-loops were enriched on 5S rRNA genes in sperms and 4-cell stage embryos, while strong sense R-loops (the R-loops in which RNA strands are the products of sense transcription) enrichment were found on 5S rRNA genes in embryos at the 256-cell and the dome stages, and in muscle cells (Figs. S6B and S6C). This trend is different from that of 45S rRNA genes, which showed high R-loop level at the 4-cell stage (Fig. 3E and Fig. S6C). The R-loops were not enriched at most lincRNA (long intergenic non-coding RNA) gene loci (Fig. S6D). However, dramatic enrichments of sense and antisense R-loops were found at miR430 loci (Figs. S6E and S6F), whose expressions are highly induced during ZGA and responsible for morphogenesis (Giraldez et al., 2006). Furthermore, analysis of the correlation between R-loops and repeats in early zebrafish embryos showed that, in general, R-loops were enriched on MSAT (Microsatellite) and LTR retrotransposons like *Copia* or *Gypsy*, but negatively correlated with some other elements like DNA transposons (Fig. S6G).

**Fig. 4:**
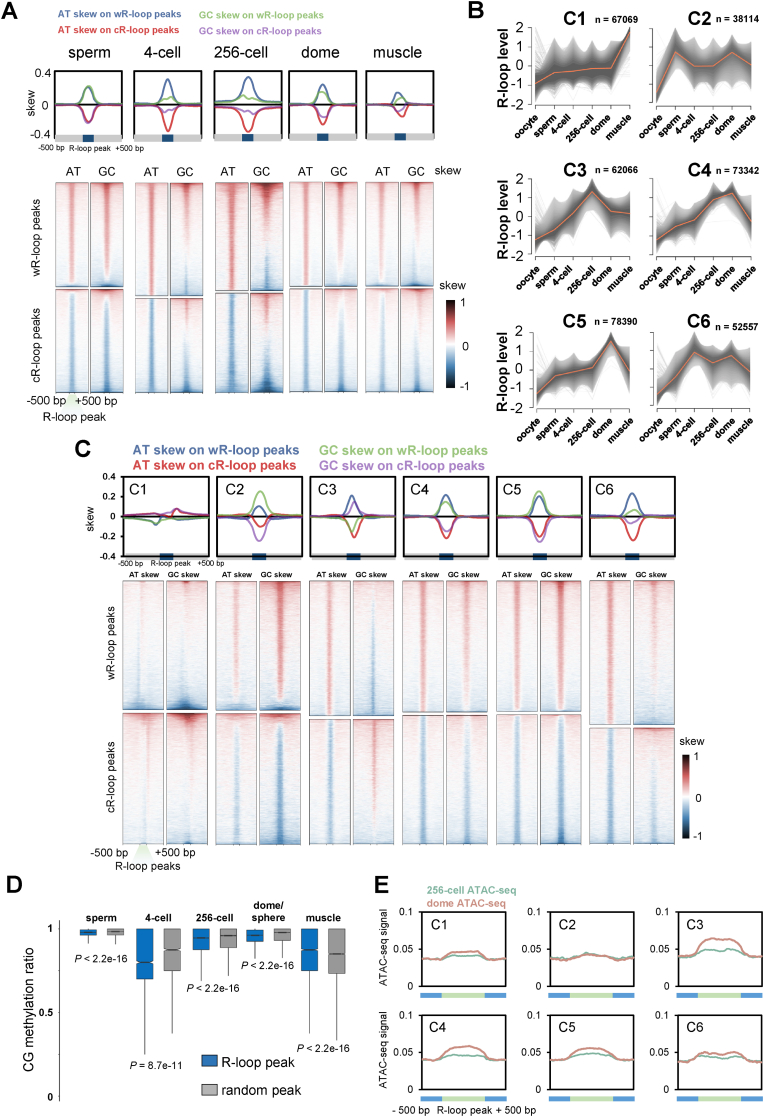
**Characteristics of R-loop dynamics during early development.** **A.** Metaplots (upper) and heatmaps (lower) of AT or GC skew around R-loop peaks of sperm, 4-cell, 256-cell, dome and muscle groups. **B.** Fuzzy cluster analysis of R-loop signals by using Mfuzz. Line plots show ULI-ssDRIP-seq signals, with individual loci (grey lines) and values of the cluster center (orange line). The count of R-loop peaks from each cluster is shown as “n”. R-loop peak locations of each cluster are shown in Table S4. **C.** Metaplots (upper) and heatmaps (lower) of AT or GC skew around R-loop peaks of clusters 1 to 6 shown in Fig. 4B. **D.** Box plot of CG methylation levels (SRP020008) on R-loop peaks (blue) or random peaks (grey) in sperm, muscle, and 4-cell, 256-cell, dome/sphere stage embryos. As sphere-stage is very close to dome stage, the CG methylation pattern at sphere stage is analyzed with R-loop peaks at dome stage. *P* values were calculated using Mann-Whitney *U* test. **E.** Metaplots of ATAC-seq signals (GSE101779) around six R-loop clusters (Fig. 4B) in zebrafish embryos at 256-cell or dome stage.

In many organisms, GC or AT skew has been reported to be a common feature of R-loops (Ginno et al., 2013; Wahba et al., 2016; Xu et al., 2017). Indeed, after calculating the GC or AT skew values on R-loop peaks from each group, we found wR-loop peaks were GC or AT skew, while cR-loop peaks were shown by negative values (Fig. 4A). In addition, we found the GC/AT skew exhibited dramatic dynamics during development (Fig. 4A). For AT skew, the summit value was increased from ∼0.2 in sperms to ∼0.3 at 4-cell and 256-cell stages, then returned to ∼0.2 at the dome stage, and decreased to ∼0.1 in muscle cells finally (Fig. 4A). For GC skew, the summit value was roughly equal with AT skew in sperm, then decreased to ∼0.1 at 4-cell and 256-cell stages, recovered slightly at the dome stage, and finally decreased to ∼0.1 in muscle (Fig. 4A).

### 2.5 Dynamic clusters of R-loop regions during early development

As dramatic changes in R-loop distributions were found during early embryonic development, we further systematically clarified the R-loop dynamics to understand the orders and characteristics of the R-loop regulatory mechanism. First, the fuzzy c-means analysis (Kumar & M, 2007) sorted R-loop regions into six clusters, from C1 to C6, and the counts of each cluster were of the same order of magnitude, as C5 contained the most R-loop regions (78390) and C2 contained the least (38114). C1 represented somatic R-loop enriched regions only (Fig. 4B). C2 represented sperm-specific R-loop enriched regions, in which the R-loop levels were highest in sperm group and second highest in dome group (Fig. 4B). C3 represented R-loop regions that showed high R-loop levels at the 256-cell stage, while C4 and C5 represented dome-specific R-loop enriched regions which showed the highest R-loop levels at the dome stage (Fig. 4B). The difference between C4 and C5 was that C4 contained additional regions which showed R-loop enrichment at the 256-cell stage (Fig. 4B). C6 represents zygotic R-loop enriched regions, which showed stable R-loop enrichments at zygotic stages only (Fig. 4B). Briefly, six major patterns of R-loop dynamic regions were defined, as C1 belonged to the somatic pattern, C2 belonged to the sperm R-loop pattern, C3 belonged to the pre-ZGA pattern, C4 belonged to the cross-ZGA pattern, C5 belonged to the post-ZGA pattern, and C6 belonged to the zygotic R-loop pattern.

As shown above, R-loop regions at different stages showed different GC or AT skew patterns. Therefore, we then analyzed the GC or AT skews in these six R-loop clusters, which indicated the various GC or AT skew patterns in different clusters (Fig. 4C). In general, C2, C4, C5, and C6 showed positive GC and AT skews (Fig. 4C), which was consistent with previous results (Ginno et al., 2013; Xu et al., 2017). Unlike the relatively high consistency of AT skew patterns among these four clusters, GC skew patterns showed more differences, such as a weaker GC skew in C6 and a stronger GC skew in C2 (Fig. 4C). Interestingly, unexpected skew patterns were found in C1 and C3 clusters. C3 showed a positive AT skew but a negative GC skew, and C1 showed both slight negative GC and AT skews (Fig. 4C). This abnormal result regarding GC/AT skew and correlation with R-loop formation suggested a possible regulatory mechanism to actively promote R-loops in the negative GC/AT skew regions, which is not suitable for thermodynamically stable R-loop formation. As C1 and C3 represented somatic and 256-cell patterns, this proportion of R-loops might be important in developmental regulation. To further characterize the R-loop clusters with abnormal GC skew, the CG methylation pattern on each R-loop cluster was analyzed. The results showed that lower CG methylation levels were found on C3 and C4 at 256-cell or dome stage (Fig. S7A). Since hypomethylation is associated with promoting chromatin accessibility and R-loop formation (Ginno et al., 2012; Liu et al., 2018), this result suggested a possible explanation of the abnormal negative GC skew on C3 R-loops, that hypomethylation might help the forming of R-loop by promoting chromatin accessibility to overcome the effect of the negative GC skew.

### 2.6 Dynamics and correlation of R-loop, DNA methylation and chromatin accessibility during early development

DNA methylation has been shown to anticorrelate with R-loop formation (Ginno et al., 2012; Lim et al., 2015). Furthermore, DNA hypomethylation correlates with other important chromatin features and regulates ZGA (Wu et al., 2021; Zhang et al., 2018). Therefore, we were curious about the correlation between R-loop and CG methylation (data from SRP020008 (Potok et al., 2013)) during early development. As shown in Fig. 4D, at the 4-cell stage, R-loop regions showed lower methylation ratio than the background (average methylation ratio), while few differences in methylation levels were found in sperms, 256-cell stage embryos, dome stage embryos, and muscles. This result suggested that the correlation between CG methylation and R-loop might occur during 4-cell stage, in which unmethylated or low-methylated regions might be apt to form R-loops. As it was shown in Fig. S7B, at the 4-cell stage, the CG methylation level in R-loop regions was lower than that in random regions, whereas no significant difference in CG methylation level between R-loop and non-R-loop regions was found at the 256-cell stage. These results suggest that, the anti-correlation between R-loop and CG methylation occurred at the 4-cell stage, rather than at the 256-cell stage. Next, we analyzed the correlations between R-loop dynamics and unmethylated regions (UMRs) or lower methylated regions (LMRs) in the genome at the 256-cell stage (Zhang et al., 2018). We found that the UMRs or LMRs showed negative or weak correlations with all clusters of R-loop regions at the 256-cell stage (Fig. S7C).

Dynamics of chromatin accessibility is another important regulatory mechanism of ZGA (Liu et al., 2018; Pálfy et al., 2020), while R-loop has been reported to be associated with hyperaccessibility region (Chedin, 2016). Thus, the correlation between R-loop and chromatin accessibility was then analyzed. The results showed that chromatin hyperaccessibility was found on R-loop peaks (Fig. 4E), which is consistent with previous reports (Chedin, 2016). Interestingly, the strongest increase of chromatin accessibility at dome stage was enriched on C3 R-loop peaks, whose R-loop level is highest at 256-cell stage (Fig. 4E and Fig. S7D). This result indicated that the R-loop level of C3 was promoted prior to the increasing in accessibility during ZGA, which suggested that a part of ZGA-related hyperaccessibility might be induced by R-loops.

Together, these results demonstrated a slight correlation between R-loop and DNA methylation but a positive correlation between R-loop and chromatin accessibility during early development.

### 2.7 Enrichment of R-loops at enhancers during ZGA

**Fig. 5:**
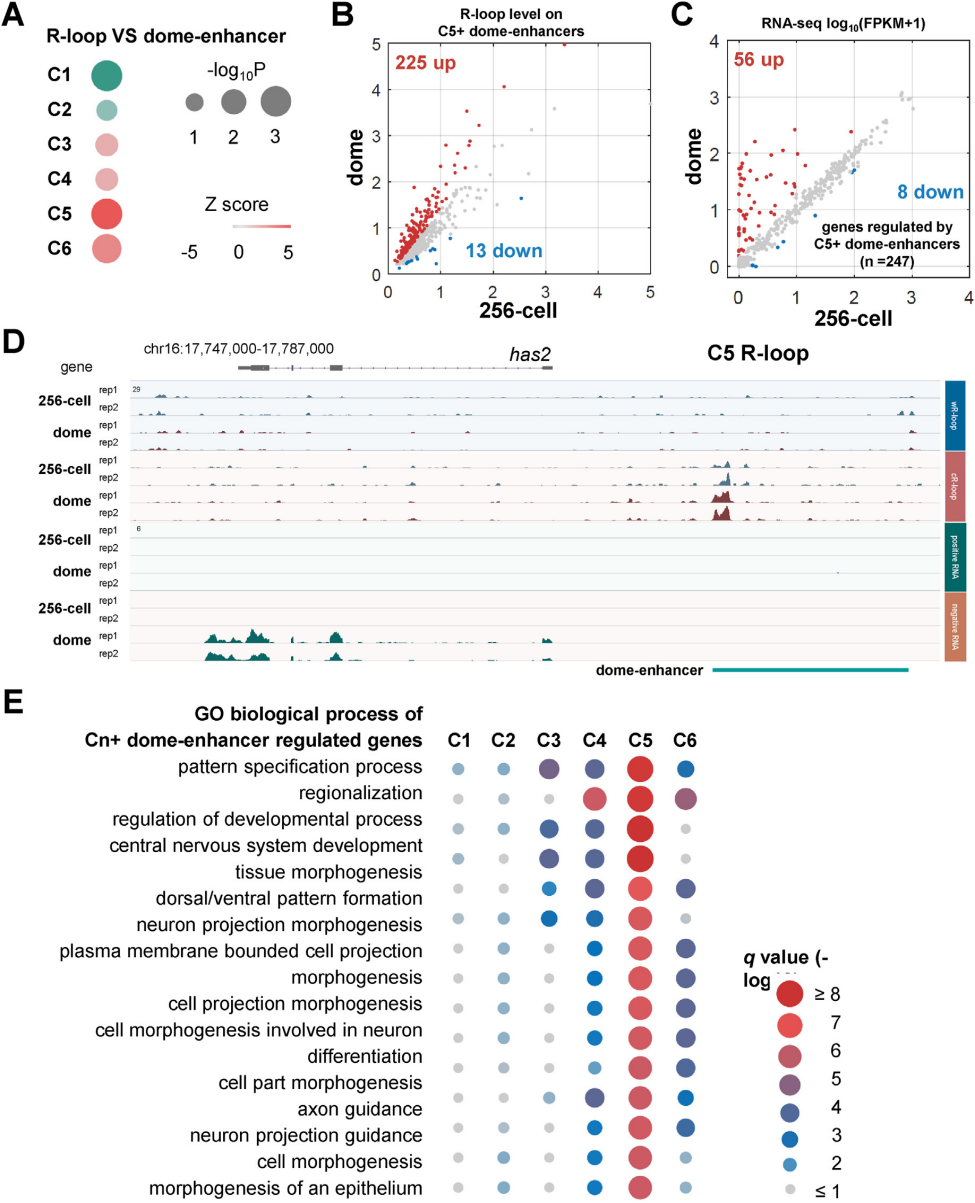
**Correlation between R-loops and enhancers during early development.** **A.** Permutation test results of correlations between enhancers at dome stage (represented as dome-enhancer, GSE114954) and six R-loop clusters shown in Fig. 4B **B.** Scatter plot of R-loop levels at 256-cell and dome stages on enhancers overlapping with C5 R-loop (represented as C5+ dome-enhancers). Up, log_2_FC > 0.5 (dome/256-cell), red. Down, log_2_FC < −0.5 (dome/256-cell), blue. **C.** Scatter plot of RNA expression levels [log_10_(FPKM+1)] of genes regulated by C5+ dome-enhancers. “Regulated” was defined as the nearest genes from enhancers. Up, log_2_FC > 1 (dome/256-cell), red. Down, log_2_FC < −1 (dome/256-cell), blue. **D.** IGV snapshot of strand-specific ULI-ssDRIP-seq (upper) and RNA-seq (lower) signals around *has2* gene at 256-cell and dome stages. C5 R-loop and dome-enhancer were shown. **E.** Gene Ontology (GO) biological process analysis results of genes regulated by dome-enhancers overlapping with each R-loop cluster (represented as Cn ​+ ​dome-enhancers). The *q* values were shown.

Pre-ZGA deposition of H3K27ac, which marks active enhancers for zygotic gene activation, plays an important role in ZGA (Zhang et al., 2018). Therefore, we wondered whether the R-loop dynamics correlate with enhancer activation during the transition from 256-cell to the dome stage. The enhancer was defined as distal genomic regions marked by H3K27ac with the absence of H3K4me3 (Creyghton et al., 2010; Zhang et al., 2018). The permutation test results indicated negative correlations between H3K27ac (data from GSE114954 (Zhang et al., 2018)) and R-loop peaks in sperm and 256-cell stage embryos, respectively, while weaker negative enrichment was found at the dome stage (Fig. S8A). Next, the correlation between H3K27ac peaks and each R-loop cluster (Fig. 4B) at 256-cell or dome stage was analyzed. Surprisingly, despite negative or weak correlations were observed between most R-loop clusters and H3K27ac peaks, C5 R-loops were strongly enriched with H3K27ac peaks at the dome stage (Fig. S8B). Furthermore, the correlation analysis showed that C5 R-loops, compared to other R-loop clusters, were strongly enriched on enhancers at the dome stage (dome-enhancers) (Fig. 5A and Fig. S8C). As C5 R-loop regions represented the highest R-loop level at the dome stage (Fig. 4B), we assumed that R-loop was enriched at enhancers during ZGA. Indeed, results from the detailed analysis confirmed this, as more dome-enhancers that overlapped with C5 R-loops showed increased R-loop levels (Fig. 5B and Fig. S8D)

The elevation of R-loop signals at dome-enhancers prompted us to investigate whether this enrichment played roles in RNA expression regulation. Thus, we checked the RNA expression levels of genes regulated by dome-enhancers that overlap with C5 R-loops (named C5+ dome-enhancers). Among those genes, 56 showed significant activation, while only eight were repressed (Fig. 5C), and the transcription rates of these 56 genes were also up-regulated during ZGA (Fig. S8E). For instance, at the *has2* locus, a zygotic gene activated at the dome stage, the increased R-loop signals were detected on its upstream enhancer region (Fig. 5D). The GO (Gene Ontology) analysis revealed that C5+ dome-enhancer-regulated genes were enriched in development-related processes, and this pattern was not found in other dome-enhancer-regulated genes (Fig. 5E), further implying the specific functions of C5+ dome-enhancer-regulated genes during ZGA.

Next, we analyzed the correlation between R-loops and enhancers in sperm. Similarly, R-loops were found to be enriched on sperm-specific enhancers (Fig. S8F). However, GO analysis of sperm-enhancer-regulated genes, classified by overlap with six R-loops clusters, showed that there was no significant functional enrichment (Fig. S8G).

Together, these results indicate that a proportion of R-loops are enriched on enhancers, and these R-loops likely promote ZGA by loading on enhancers.

### 2.8 Enhancer R-loops promote zygotic gene expression

Given that the correlation between R-loops and enhancers existed as shown above, we wondered whether the R-loops enriched on enhancers (termed enhancer R-loops) play any roles in enhancer-induced gene expression regulation. In the previous study in plants, we had developed a dCas9-RNH1 system with “dead” Cas9 (dCas9) fused with Arabidopsis RNaseH1 (AtRNH1C) to decrease the levels of R-loops in a locus-specific manner (Liu & Sun, 2021). RNaseH1 has been reported to be involved in global R-loop elimination (Santos-Pereira & Aguilera, 2015). Here, we modified this *dCas9-hRNaseH*-based R-loop modulation system by replacing *AtRNH1C* with human *RNaseH1*, which has been reported to be involved in global R-loop elimination (Cerritelli & Crouch, 2009), and applied it in the zebrafish (Fig. S9A). A *dCas9-hRNaseH1*^D210N^ (D210N mutant, lacks endonucleolytic activity) was used for negative control (Fig. S9A). Two zygotic genes, *sox3* and *has2* were chosen from 56 genes which showed a positive correlation between enhancer-R-loop and RNA expression (Fig. 5C). Two guide RNAs (gRNAs), which target the flanks of R-loop peaks at enhancers in the upstream of *sox3* and *has2* genes, were designed to reduce the R-loop levels (Figs. S9B and S9C). After injection of *dCas9-hRNaseH1* mRNA and gRNA targeting *sox3* enhancer-R-loop at the 1-cell stage, the RNA expression level of *sox3* at the dome stage was significantly decreased compared with that in samples without gRNA injection (Fig. S9D). Combined with the result that co-injection of *dCas9-hRNaseH1*^D210N^ mRNA and gRNA did not change the expression level of *sox3*, these results suggest that the expression of *sox3* gene was due to the R-loop formation at its upstream enhancer (Fig. S9D). A similar result was found at the *has2* locus (Fig. S9D). Together, disruption of enhancer R-loops reduced the expression levels of *sox3* and *has2*, suggesting a positive role of enhancer R-loops in promoting zygotic gene expression.

### 2.9 Balanced R-loop dynamics ensures normal embryonic development

**Fig. 6:**
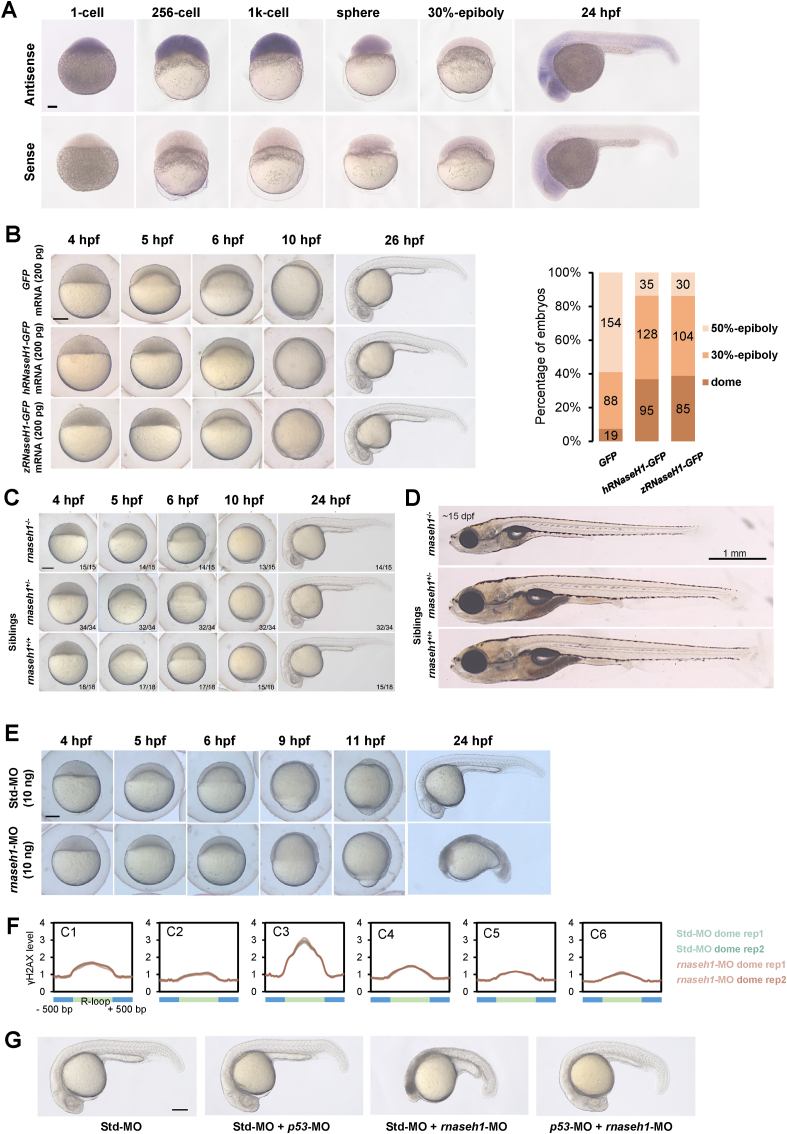
**Balanced R-loop dynamics ensures active zygotic gene expression and secure early embryonic development.** **A.** Spatiotemporal expression pattern of *rnaseh1*. Embryos were laterally viewed with animal pole to the top or with anterior to the left. Scale bars, 100 μm. **B.** Left, representative embryos from *GFP*-injected, *hRNaseH1-GFP*-injected and *zRNaseH1-GFP*-injected groups. Scale bars, 200 μm. Right, the ratio of embryos with different development stages at 5.5 hpf. **C.** Representative embryos of *rnaseh1* homozygous mutants with siblings at early development stages. The ratio of zebrafish with the similar results is indicated. Scale bars, 200 μm. **D.** Representative fish of *rnaseh1* homozygous mutants with siblings at 15 dpf. Scale bars, 1 mm. **E.** Representative embryos from 10 ng Std-MO-injected and 10 ng *rnaseh1*-MO-injected groups. Scale bars, 200 μm. **F.** Metaplots of γH2AX stacc-seq signals around six R-loop clusters (Fig. 4B) in Std-MO and *rnaseh1*-MO embryos at dome stage. **G.** Morphology of *rnaseh1*-knowdown embryos coinjected with *p53*-MO compared to the control embryos at 24 hpf. Scare bar = 200 μm.

As dramatic changes in R-loops occurred during embryogenesis (Fig. 4), we wondered whether artificial modulation of global R-loops might affect normal development of early embryos. To verify the expression pattern of *rnaseh1* in zebrafish embryos, we performed RNA whole-mount *in situ* hybridization. Zebrafish *rnaseh1* mRNA was maternally expressed and exhibited ubiquitous expression during early blastula stage, and gradually degraded after sphere stage (Fig. 6A). At 24 hpf, the mRNA was mainly expressed in the head and tail (Fig. 6A). To decrease global R-loops, *hRNaseH1* mRNAs were injected at 1-cell stage, while *GFP* and catalytically inactive *hRNaseH1*^D210N^ were used as negative controls (Fig. S10A). The immunofluorescence results using S9.6 antibody showed that R-loop signals in the nucleus were significantly decreased after *hRNaseH1* mRNA injection compared with those in the *GFP* control, while slight signal enhancements were found in *hRNaseH1*^D210N^ injection group (Figs. S10A and B). The ULI-ssDRIP-seq result also showed the down-regulation of R-loop signals after overexpression of hRNaseH1 (Fig. S10C), and the R-loop levels on the two dome enhancers were decreased in + *hRNaseH1-GFP* dome (Fig. S10C). These results demonstrated that *hRNaseH1* injection could reduce R-loop level globally, which might affect the process of parental-to-zygotic transition. The development processes of injected embryos were then observed persistently, and the results showed a developmental delay after sphere stage in *hRNaseH1* injection group (Fig. 6B). The ratio of embryos with different developmental stages were counted at 5.5 hpf, and the result showed a slower epibolic process of embryos injected with *hRNaseH1* mRNA (Fig. S10D). Injection of zebrafish *rnaseh1* mRNA also resulted in a similar phenotype with *hRNaseH1* injection (Fig. 6B). These results indicated that global reduction of R-loops could disrupt the developmental process of embryos, suggesting that R-loop dynamics is necessary for normal embryogenesis.

As parental-to-zygotic transition requires proper activation of the zygotic genome, we then analyzed the transcriptomes of *hRNaseH1* and *GFP* injected groups at the dome stage by using RNA-seq. Our results showed that, 2802 differential expression genes (DEGs) were found, and 1124 of them were up-regulated while 1678 of them were down-regulated after *hRNaseH1* injection (Fig. S10E, Table S5). Among them, about 52% of up-regulated DEGs were maternal genes, while 55% of down-regulated DEGs were zygotic genes (Figs. S10E and S10F)(Lee et al., 2013), which might be the cause of embryonic developmental delay. It was also shown that the ratio of unchanged genes without R-loops was much higher than those with R-loops (Fig. S10G), suggesting the R-loop formation was helpful for regulating gene expression.

Furthermore, we wondered whether artificially modulating the changes of global R-loop could affect the embryonic development process or not. A *rnaseh1* mutant line was generated using the CRISPR/Cas9 system. The mutation resulted in a 5-bp deletion and the introduction of a premature stop codon behind (Fig. S11A). Despite the morphology of *rnaseh1* mutants appearing nearly normal until 6 days post-fertilization (dpf), the body length of homozygous mutants was significantly shorter than siblings with the head developed abnormally (Fig. 6C and D and Fig. S11B). Moreover, *rnaseh1* homozygotes died within 7–21 dpf (Fig. S11C). As no obvious phenotypic differences were found during early development (Fig. 6C), we suspected that maternal expression of *rnaseh1* might protect R-loop stability in early stage embryos.

Since *rnaseh1* homozygotes were lethal, it is unable to obtain the maternal mutants. The morpholino (*rnaseh1*-MO) injection was performed, which could effectively inhibit mRNA translation to knock down *rnaseh1* expression level (Fig. S12A). It was found that the R-loop level was up-regulated consequently (Fig. S12B) and *rnaseh1* knockdown caused a delay in embryo epibolic process compared with injection of the same dose of control MO (Fig. 6E and Fig. S12C). The ratios of embryos with different developmental stages were counted at 5 hpf and 6 hpf, and the result showed a much slower epibolic process of embryos injected with *rnaseh1*-MO (Fig. S12D). We also found that MO injection could induce head and tail apoptosis at 24 hpf in a dose dependent manner (Fig. S12C). Considering that the cause of developmental delay might be related to DNA damage, we detected total γH2AX level at dome stage and found there was no significant difference between the groups injected with *rnaseh1*-MO or control MO (Fig. S13A). Furthermore, genome-wide DNA damage was profiled by using γH2AX stacc-seq. The results showed that γH2AX signal at dome stage was enriched at R-loop loci, especially at C3 cluster loci which represent 256-cell-specific R-loop (Fig. 6F), suggesting that R-loop might regulate ZGA via regulating DNA damage. However, few differences in γH2AX stacc-seq signal were found between *rnaseh1*-MO and control (Fig. 6E and Fig. S13B and S13C). Therefore, we believed that the developmental delay by *rnaseh1* knockdown was not due to DNA damage changes. Besides, the RNA-seq results showed that more maternal genes were activated in *rnaseh1*-MO group (Fig. S13D). As both up- and down-regulation of *rnaseh1* could delay embryogenesis, we then compared the expression changes induced by *rnaseh1* know-down and overexpression. The results showed 268 common up-regulated DEGs and 141 common down-regulated DEGs (Fig. S13E). Notably, GO results showed that the common down-regulated DEGs were enriched in endoderm development, which might be involved in the embryogenesis delay (Fig. S13E). Together, the observed developmental defects caused by R-loop alterations suggested that R-loop dynamics balances early embryonic development and parental-to-zygotic transition in the vertebrates.

To determine whether the developmental defects observed in *rnaseh1*-MO injection group were due to apoptosis, we analyzed the embryos using a TUNEL assay. The increase in TUNEL positive signal was observed in the knock-down embryos at 24 hpf (Fig. S14A). In contrast, the control MO injected embryos showed only a few scattered apoptotic cells. To investigate whether a *p53*-pathway was activated with the loss of *rnaseh1*, which then caused head and tail apoptosis, we analyzed the expression level of *p53* and its target genes in *rnaseh1*-MO injected embryos at 5 hpf and 12 hpf. At 5 hpf, there was no difference in the transcript levels of p53 between *rnaseh1* knock down and control embryos (Fig. S14B). However, by 12 hpf, marginal upregulation of *p53* transcripts were evident in *rnaseh1* knock-down embryos. The proapoptotic Bcl-2 family genes (*bik* and *bax*), which are regulated by *p53*, also showed transcriptional upregulation in the *rnaseh1* knock down embryos at 12 hpf (Fig. S14C). The prosurvival markers (*blp1* and *mcl1a*) were not significant changed, indicating an activated apoptotic pathway, which was consistent with the TUNEL results (Fig. S14C). Since the *rnaseh1* knock-down activates a p53-dependent apoptotic response, we examined whether simultaneous knockdown of *p53* and *rnaseh1* could rescue the developmental defects and apoptosis of *rnaseh1* knock-down embryos. We coinjected *rnaseh1*-MO (2 ng) and *p53*-MO (2 ng), or their respective control MOs, into zebrafish at the one-cell stage, and analyzed the resulting phenotypes. Coinjection of *rnaseh1*-MO and *p53*-MO could partly rescue the developmental defects (Fig. 6G). In summary, these results showed that upregulation of R-loops induced by *rnaseh1* knock-down during embryogenesis would activate a *p53*-dependent apoptosis and cause the apoptotic phenotype after the later stage of segmentation period.

## 3 Discussion

The regulatory mechanisms of zygotic gene activation during early embryonic development have been studied at multiple levels of epigenetics, including DNA methylation and histone modification (Liu et al., 2018; Schulz & Harrison, 2019; Zhang et al., 2018). As R-loop is considered to be a new player in genome regulation (Crossley et al., 2019; Petermann et al., 2022; Santos-Pereira & Aguilera, 2015), we were then promoted to investigate its features and biological functions during ZGA. By introducing mung bean nuclease pretreatment step and optimizing the experimental process, we have developed the ULI-ssDRIP-seq that is suitable for detecting R-loops from ultra-micro samples.

Mung bean nuclease was the key step in ULI-ssDRIP-seq, not only because it can improve the sensitivity and boost the resolution to near-single-nucleotide level (Fig. 1), but also because it can eliminate excessive free RNAs, which was common in egg cells and very early embryos (Olszanska & Borgul, 1993). By pre-ligation of adapters, the steps for DNA extraction were greatly reduced (Fig. 2A), the experimental process was obviously shortened in ULI-ssDRIP-seq, and RNA:DNA hybrids were maximally retained. Different from qDRIP-seq (Crossley et al., 2020), a quantitative R-loop profiling method using annealing RNA:DNA hybrids from *in vitro* transcription products and DNA templates as the spike-in, ULI-ssDRIP-seq uses gDNA from a distant species for quantification, which can provide inter-sample and inter-species quantitative information of total R-loop levels simultaneously. In addition to this study in embryonic development, ULI-ssDRIP-seq could also be reaching to other special samples containing rare cells, such as some tiny pathological tissues, environmental microorganisms, specific types of immune cells, or deeply sorted cells. This will certainly provide some novel insights for the relevant biological research interests.

In this study, we have generated a comprehensive R-loop atlas of zebrafish gametes and early embryos, and provided a wealth of information for investigating R-loop dynamics and functions. The first interesting finding was that strong R-loop enrichments were located on the maternal 45S rDNA gene in zebrafish oocytes, while rare R-loops were found in other regions of nuclear genome (Fig. 3D and E and Fig. S6C). This might be due to the special mechanism of maternal/somatic type switch of 45rRNA during the parental-to-zygotic transition in zebrafish. Whether this enrichment of R-loops on maternal 45S rDNA gene has a specific biological function requires further investigations.

Another interesting phenomenon was the negative GC skew of a proportion of R-loops (Fig. 4C), which has not been reported in previous studies. As reported previously, superior thermodynamic stability was found in the RNA:DNA hybrid of G-rich RNA and complementary C-rich DNA (Roberts & Crothers, 1992). Therefore, these low-stability R-loops may be generated by unknown active regulation mechanisms. In addition, such data are also in line with the S9.6 antibody minor groove recognition of RNA:DNA hybrids (Bou-Nader et al., 2022; Li et al., 2022), rather than the sequence preference. The weak correlation between R-loops and DNA methylation during early development is unexpected, enlightening us that formation and regulatory mechanisms of R-loops could be multifarious.

The R-loop level in a group of regions, C5 R-loops (Fig. 4B), increased dramatically during ZGA, which might be due to the repression of *rnaseh1*, an R-loop eraser that hydrolyzes the RNA from an RNA:DNA hybrid (Figs. S3B and 6A). Interestingly, C5 R-loops were enriched on ZGA-specific enhancers (Fig. 5A), and we further showed the evidence that the enhancer R-loops are necessary for activation of the zygotic genes *sox3* and *has2* (Fig. S9). eRNA (enhancer RNA), a group of short noncoding RNA transcribed from the enhancer regions, is an important functional component in the enhancer complex (Kim et al., 2010) and previous results had shown that eRNA formed R-loop (Pefanis et al., 2015; Zhou et al., 2021). However, the direct evidences to show the function of enhancer R-loop activity on modulating gene expression are missing. Based on the *dCas9-hRNaseH1* system in this study, we proved that enhancer R-loops may play a key role in activating zygotic genes during ZGA (Fig. 5 and Fig. S9).

The phenotypic results also showed global increase or decrease in R-loop levels could both delay embryogenesis (Fig. 6), suggesting that the balance of R-loops is essential for embryogenesis. Transcriptomic results also demonstrated that the process of ZGA is dramatically affected (Fig. 6). These results suggest that a ZGA-balanced R-loop dynamics could secure the zygotic gene activation, and thus preserve parental-to-zygotic transition for proper early embryonic development. Notably, apoptosis induced by R-loop promotion during embryogenesis is dependent on the p53 pathway, providing novel insights into the relationship between R-loop and apoptosis. As the steps and regulation of embryogenesis in different species vary enormously, inspired by this study, we believe novel biological functions of R-loops during embryogenesis will be uncovered in the future.

## 4 Materials and methods

### 4.1 Animal materials and culture methods

Zebrafish Tübingen (TU) line was maintained in the Meng Lab. The laboratory animal facility was accredited by AAALAC (Association for Assessment and Accreditation of Laboratory Animal Care International), and the IACUC (Institutional Animal Care and Use Committee) of Tsinghua University approved all animal protocols used in this study.

Fish were maintained at 28.5 °C. One female and one male fish were separated in a mating tank at night and allowed their mating by removing the separator next morning at a desired time, followed by collecting and incubate fertilized eggs to desired stages. Mature eggs (stage V oocytes) and sperms were squeezed from female and male, respectively.

To exclude the high amount of free RNA and mitochondrial DNA in mature eggs and 4-cell stage embryos, crude nuclei were fractionated before genomic DNA extraction. High quality eggs were first collected in 1% BSA in Hank's solution without calcium and magnesium (1% BSA, 0.137 M NaCl, 5.4 mM KCl, 0.25 mM Na_2_HPO_4_, 0.44 mM KH_2_PO_4_, 4.2 mM NaHCO_3_) (Potok et al., 2013) until enough. Then eggs were activated by immersion in Holtfreter's solution (59 mM NaCl, 0.67 mM KCl, 0.76 mM CaCl_2_, 2.4 mM NaHCO_3_, pH 6.9–7.3) for 10 min. The chorion was removed by Pronase (Sigma) treatment at 28.5 °C for approximately 10 min. Then eggs were washed thoroughly in Holtfreter's water in a plate, then transferred to a 1.5-ml Eppendorf tube. 1 ml of chilled cell lysis solution (10 mM Tris-Cl [pH 8.0], 10 mM NaCl, 0.5% NP-40) was added to each 500 eggs, then eggs were broken with a 21-gauge needle on ice. Nuclei were spun down at 3500 ×*g* for 5 min at 4 °C. The nuclei of 4-cell stage embryos were collected with the same process from the step of Pronase treatment.

Semen collected in the capillary tube was transferred to an Eppendorf tube containing 50 μl Hank's solution (1% BSA, 0.137 M NaCl, 5.4 mM KCl, 0.25 mM Na_2_HPO_4_, 0.44 mM KH_2_PO_4_, 4.2 mM NaHCO_3_, 1.3 mM CaCl_2_, 1 mM MgSO_4_), then subjected to genomic DNA extraction.

For 256-cell stage and dome stage embryos, whole embryos were used. Naturally spawned fish embryos were collected at the one-cell stage and cultured in Holtfreter's solution to indicated stages.

For muscle tissue, euthanasia of male fish was carried out by immersing it in ice fish water for 10–15 min until the fish stopped breathing. The fish scales, skin, head, fins, and internal organs were removed, and the body was washed with cold PBS several times. The muscle tissue on the backside of the fish was cut off and transferred to an Eppendorf tube for genomic DNA extraction.

The approximate starting material for each sample replicate: 3000–4000 mature eggs, approximately 1000∼2000 4-cell stage embryos, 600–800,256-cell or dome stage embryos, semen from 2 male fish.

### 4.2 Genomic DNA extraction

Samples collected were lysed in genome extraction buffer (200 mM NaCl, 10 mM Tris-HCl pH 8.0, 10 mM EDTA, 0.5% SDS, 200 μg/ml freshly added Proteinase K) at 37 °C for 4–12 h. Then, a 1/4 volume of 5 M potassium acetate solution (pH 5.2) was added to the lysate and mixed gently, followed by incubation on ice for 20 min. Following extraction with an equal volume of phenol: chloroform: isoamyl alcohol (25:24:1, pH 7.8) and chloroform sequentially with phase-lock tubes. The supernatant containing genomic DNA was precipitated by addition of an equal volume of isopropanol and 1 μl GlycoBlue (Thermo Fisher) for 12 h at −20 °C. The precipitate was washed with 70% ethanol once and then air-dried. The negative control was treated with 10 U RNase H (New England Biolabs) at 37 °C overnight.

### 4.3 Basic or modified ssDRIP-seq

Basic ssDRIP-seq was performed as described previously (Xu et al., 2017; Yang et al., 2019). *Dde*I, *Mse*I, *Nla*III, and *Mbo*I were used for gDNA fragmentation in Arabidopsis, while *Mbo*I, *Mse*I, *Dde*I and *Alu*I were used in zebrafish. To pursue higher resolution and lower minimum input, a modified ssDRIP-seq was developed. For modified ssDRIP-seq, gDNA from 7-day-old seedlings of Arabidopsis (Col-0) was digested by S1 nuclease (Thermo Fisher; final concentration: 5 U/100 μl), dsDNA Fragmentase (New England Biolabs; final concentration: 5 U/100 μl), dsDNase (Thermo Fisher; final concentration: 5 U/100 μl), or mung bean nuclease (TaKaRa; final concentration: 5 U/100 μl) overnight at 37 °C instead of restriction enzymes used in basic ssDRIP-seq. After purification by the phenol-chloroform method, digested gDNA was DRIPed and used for ssDNA library construction as described previously (Xu et al., 2017).

### 4.4 Nuclease efficiency assay

To prepare half R-loop substrates, RNA (5′-GGGAUGGUGCUGGACUCAUUCGGCAUCGGCGCUACAGAAGAUGCAGAACGCUUUGGUGACGUCGGGGCUGACACCCUGGGUCAUAUCGCAGAAGCUUGUGCCAAAGGCGAAGCUGAUAACGGUCGUAAAGGCCCGCUCAAUCUGCCAAAUCUGACCCGUCUGGGGCUGGCGAAAGCACACGAAGGUUCUACCGGUUUCAUUCC-3′) was produced by using *in vitro* transcription kit (New England Biolabs; M0658) and purified with phenol-chloroform method. In the following reverse transcription step, PCR templates was removed by gDNA digester (YEASEN; 11141ES10), followed by cDNA synthesis with primer (5′-ACGTGTCATGACTGACACTGGCAGTACGTAGCAGTGGTAGAACCTTCGTGTGC-3′) and paired DNA oligo (5′-TTCTACCACGGAACTGCTACGTACTGCCAGTGTCAGTCATGACACGT-3′). following the protocol (YEASEN; 11141ES10). The artificial R-loop was purified by 2 vol of SPRIselect beads. 10 ng purified artificial R-loop was mixed with 20 U mung bean nuclease, S1 nuclease, dsDNase, dsDNA Fragmentase respectively, and incubated at 37 °C for 10 min. The digested product was purified using 1.8 vol of SPRIselect beads. Purified product was DRIPed and used for ssDNA library construction as described previously (Xu et al., 2017). The library was sequenced on an Illumina NovaSeq system.

### 4.5 ULI-ssDRIP-seq

Genomic DNA was digested with mung bean nuclease (TaKaRa) at 37 °C for 2 h and purified by the phenol-chloroform method followed by resuspending the pellet in 130 μl TE buffer. Digested gDNA was sonicated to a peak fragment size of 250 bp, performed on an S220 Focused-ultrasonicator with 10% duty factor, 200 cycles/burst, 175 peak incident power, and for 90 s per tube. For inter-group relative quantification, gDNA from Arabidopsis (7-day-old Col-0 seedlings) was used as the spike-in sample to normalize total R-loop levels between animal samples. Spike-in DNA was sonicated to a peak fragment size of 250 bp as described above, and then ∼0.1 ng sonicated spike-in gDNA was added to sonicated target gDNA samples. 80% of the mixed sample was used for the first adapter ligation of ULI-ssDRIP-seq workflow, and the remaining 10% part was used for input-library preparation by using Accel-NGS 1S Plus DNA Library Kit following the manual.

For the first adapter ligation (pre-ligation), 53 μl sonicated gDNA was added to Adaptase reaction mix (for each sample: 8 μl buffer G1, 8 μl reagent G2, 5 μl reagent G3, 2 μl enzyme G4, 2 μl enzyme G5, and 2 μl enzyme G6, from Accel-NGS 1S Plus DNA Library Kit), incubated at 37 °C for 1 h. Then DRIP was performed as described previously (Xu et al., 2017). 174 μl pre-mixed extension reaction mix was prepared following the extension part of the manual (Accel-NGS 1S Plus DNA Library Kit) and mixed with beads/antibody complexes, followed by 1 cycle PCR (98 °C 90 s, 63 °C 30 s, 68 °C 5 min). DNA was purified by adding 1.2 vol of SPRIselect beads. The second truncated adapter was ligated to the 5’ ends at 25 °C for 1 h following the ligation part of manual (Accel-NGS 1S Plus DNA Library Kit). DNA was purified again by 1 volume of SPRIselect beads and eluted with 21 μl Low EDTA buffer.

1 μl purified DNA was used for qPCR by using indexing primers to address the cycle numbers of indexing PCR. The cycle numbers of indexing PCR were equal to the Ct value. An indexing PCR step was performed following the manual of Accel-NGS® 1S Plus DNA Library Kit.

All the libraries, including basic or modified ssDRIP-seq, libraries of mung bean nuclease efficiency assay, and ULI-ssDRIP-seq were then checked on an Agilent BioAnalyzer, followed by sequencing on an Illumina NovaSeq system. Paired-end 150 (PE150) sequencing was used for ULI-ssDRIP-seq. Information of samples was list in Table S1.

### 4.6 RNA-seq

At desired developmental stages, the embryos were dechorionated by treatment with Pronase (Sigma-Aldrich). 50 embryos were collected for each sample. The dechorionated embryos were transferred to a 1.5-ml Eppendorf tube, then were lysed in 1 ml of TRIzol reagent (Thermo Fisher). Fully lysed samples in TRIzol reagent were centrifuged at 12,000 rpm for 10 min at 4 °C, the supernatant was kept and sent to Novogene for RNA-seq. RNA-seq libraries were prepared by using NEBNext Ultra RNA Library Prep Kit following manufacturer's recommendations, and sequenced on an Illumina NovaSeq 6000 platform to generated 150 bp paired-end reads.

### 4.7 γH2AX stacc-seq

γH2AX stacc-seq was performed using anti-phospho-Histone H2A.X (Ser139) antibody (Millipore, JBW301) as described previously (Liu et al., 2020).

### 4.8 Sequencing data processing

For basic or modified ssDRIP-seq, reads were aligned to the Arabidopsis TAIR10 genome with Bowtie 2 (Langmead & Salzberg, 2012) (version 2.3.4.2) using default settings. For ULI-ssDRIP-seq without spike-in, reads were aligned to the zebrafish danRer7 genome with Bowtie 2. Duplicates were removed by Picard tools (http://broadinstitute.github.io/picard). Using samtools (Li et al., 2009) (version 1.3), unmapped reads and reads with more than three mismatches were removed, and the total mapped reads (unstranded R-loops) were divided into wR-loop and cR-loop reads (wR-loop means an R-loop with a Watson strand unpaired ssDNA, while cR-loop means an R-loop with a Crick strand unpaired ssDNA) as described previously (Xu et al., 2017, 2020).

R-loop peak calling was performed by using MACS2 (Feng et al., 2012)(version 2.0.10) with default parameters, and small peaks which size <200 bp were discarded.

Aligned read files (BAM) were converted to normalized coverage files (bigWig) by using bamCoverage from deepTools (Ramirez et al., 2014) (version 2.4.2). Read coverage of data from basic or modified ssDRIP-seq, or ULI-ssDRIP-seq without spike-in was normalized to 1 × sequencing depth (also known as Reads Per Genomic Content, RPGC) with the following parameter to exclude the influences from the various copy numbers of mitochondrial DNA: ignoreForNormalization chrM.

For quantitative ULI-ssDRIP-seq, alignment and normalization steps were different from those described above. Reads from ULI-ssDRIP-seq or input library were aligned to the zebrafish danRer7 or Arabidopsis TAIR10 genome with Bowtie 2 respectively. Normalization method is briefly described in the results section (Fig. 3A). First, read count of ULI-ssDRIP-seq or input library mapped to danRer7 (target sample [T]) or TAIR10 was calculated. (spike-in sample [S]). Tn or T(n) represents target n, while Sn or S(n) represents spike-in n (n = 1, 2, 3 …, represent each sample). Any sample was chosen as an inter-sample normalization control (represented by T1; in this study, muscle rep1 was chosen as T1), and normalization factor of reads mapped to danRer7 was calculated in the RPGC manner described above. Quantitative factor was calculated as shown in Fig. 3A. T(n)_R-loop_ represents read count of ULI-ssDRIP-seq library from T(n) mapped to danRer7, while S(n)_input_ represents read count of input library from S(n) mapped to TAIR10. By analogy, T(n)_input_ represents read count of input library from T(n) mapped to danRer7, while S(n)_R-loop_ represents read count of ULI-ssDRIP-seq library from S(n) mapped to TAIR10. The quantitative factor of Tn equaled [T(n)_R-loop_ × S(n)_input_]/[S(n)_R-loop_ × T(n)_input_]. The final normalization factor of Tn equaled Quantitative factor (Tn) multiplied by Normalization factor (T1) divided by Quantitative factor (T1). For other samples except for T1, the final normalization factor was used for normalization to convert bigWig file.

Snapshots were generated by using the Integrative Genomics Viewer (Thorvaldsdottir et al., 2013) (IGV). Spearman's or Pearson's correlation coefficients were calculated with plotCorrelation from deepTools. Heatmaps and metaplots were generated with computeMatrix from deepTools. Scatter plots were drawn by using MATLAB or R scripts.

GC or AT skew was calculated as: follows: GC skew = (count of G – count of C)/(count of G ​+ ​count of C), and AT skew = (count of A – count of T)/(count of A ​+ ​count of T), with a 200 bp sliding window and a 50 bp step.

By using Mfuzz (Kumar & M, 2007), fuzzy cluster analysis was performed. All R-loop peaks studied were merged to a total peak set by using Bedtools (Quinlan & Hall, 2010). Read count from each sample on total peaks was calculated by with multiBamSummary from deepTools.

Gene ontology analysis was performed by using Metascape (Zhou et al., 2019). Peak annotation was performed by using ChIPseeker (Yu et al., 2015) (version 1.14.1). Permutation test was performed by using regioneR (Gel et al., 2016) (version 1.24.0). The annotations of transposable elements and tandem repeats were downloaded from Dfam (Storer et al., 2021) (https://dfam.org).

For RNA-seq data processing, raw reads were trimmed by TrimGalore (version 0.6.7) (https://github.com/FelixKrueger/TrimGalore) and aligned to the zebrafish genome (danRer7) using HISAT2 (version 2.2.1) with default parameters (Pertea et al., 2016). Then, raw counts for the feature of genes were extracted by featureCounts (version 2.0.3) (Liao et al., 2014). To identify differentially expressed genes (DEGs), R package edgeR (version 3.32.) were applied to organize, filter and normalize the data. Quasi-likelihood F-tests were performed to identify DEGs according to the guide (Lun et al., 2016; Robinson et al., 2010).

For γH2AX stacc-seq data processing, reads were aligned to the zebrafish danRer7 genome with Bowtie 2. Aligned read files (BAM) were converted to normalized coverage files (bigWig) by using bamCoverage from deepTools with normalization to 1 × sequencing depth (RPGC).

### 4.9 DRIP-qPCR

DRIP-qPCR was performed as described previously (Xu et al., 2017), with two biological replicates and three technical replicates for each sample. Primers used in this study are listed in Table S2.

### 4.10 Microinjection, locus-specific R-loop editing, immunofluorescence and TUNEL

For *hRNaseH1-GFP* expression experiment, injection doses are given in the figures or figure legends. The developmental stages were visually inspected and pictured under a stereomicroscope (Nikon SMZ1500). Immunofluorescence in zebrafish embryos were performed essentially as described (Chen et al., 2021). Anti-GFP antibody (1:200 dilution; Santa Cruz Biotechnology, sc-9996), anti-Cdh1 antibody (1:200 dilution; AnaSpec, 55615) and anti-S9.6 antibody (1:200 dilution; Sun Lab) were used for immunofluorescence. Embryos were observed by confocal microscopy (Olympus FV3000). For the morpholino (MO)-mediated knockdown experiments, *rnaseh1* MO (*rnaseh1*-MO, 5′-AAAGTTTCCCTTCTTGCCCATCTCT-3ʹ), *p53* MO (p53-MO, 5′-GCGCCATTGCTTTGCAAGAATTG-3ʹ) and control MO (Std-MO, 5ʹ-CCTCTTACCTCAGTTACAATTTATA-3ʹ) were purchased from GeneTools. MOs were injected into the yolk of zebrafish embryos at 1-cell stage. The injection doses are given in the figures. For the MO efficiency testing experiment, zebrafish *rnaseh1* were cloned into a pCS2+ vector with HA tag. mRNA was transcribed using the mMESSAGE mMACHINE™ SP6 kit (Invitrogen, AM1340) and injected into zebrafish embryos at 1-cell stage with MO together. The expression levels of *rnaseh1-HA* were detected with regular Western blot procedure using anti-HA (1:10,00 dilution; Santa Cruz Biotechnology, sc-7392) and α-Tubulin antibody (1:5000 dilution; GeneTex, GTX628802). Signals were detected by reaction with BeyoECL (Beyotime, P0018M) and visualized on the Imaging System (Tanon, 5200). Phospho-Histone H2A.X (Ser139) antibody (Cell Signaling Technology, 9718s) and Histone H3.1 antibody (Abmart, P30266) were used for DNA damage detection.

For detection of apoptotic cells in whole embryos, a TUNEL assay was performed using an In Situ Cell Death Detection Kit, Fluorescein (Roche, Germany).

### 4.11 Whole mount *in situ* hybridization (WISH)

Zebrafish embryos that reached the desired stages were fixed in 4% paraformaldehyde. Digoxigenin-labeled antisense/sense RNA probes were generated with T7 RNA polymerases (Roche) according to the orientation of cDNA insert. WISH was performed essentially as before (Chen et al., 2021).

## Data availability

All the NGS data and processed files in this study are available in NCBI's Gene Expression Omnibus under accession number GSE183453. Information of software used in this study is listed in Table S3.

Other reported data. RNA-seq, GSE114954. DNA methylation, SRP020008. ATAC-seq, GSE101779.

## CRediT authorship contribution statement

**Wei Xu:** Writing – review & editing, Writing – original draft, Project administration, Methodology, Data curation. **Xin Liu:** Writing – review & editing, Resources, Conceptualization. **Jinjin Li:** Validation. **Changbin Sun:** Validation, Data curation. **Luxi Chen:** Resources. **Jincong Zhou:** Resources. **Kuan Li:** Data curation. **Qin Li:** Resources. **Anming Meng:** Writing – review & editing, Project administration. **Qianwen Sun:** Writing – review & editing, Writing – original draft, Project administration.

## Declaration of competing interest

The authors declare that they have no known competing financial interests or personal relationships that could have appeared to influence the work reported in this paper.
