# Supplementary material for: ULI-ssDRIP-seq revealed R-loop dynamics during vertebrate early embryogenesis
Source: Cell Insight. 2024 Jun 1;3(4):100179. doi: 10.1016/j.cellin.2024.100179 (PMC11225018; doi:10.1016/j.cellin.2024.100179)
Supplement: Multimedia component 1 [file mmc1.pdf]

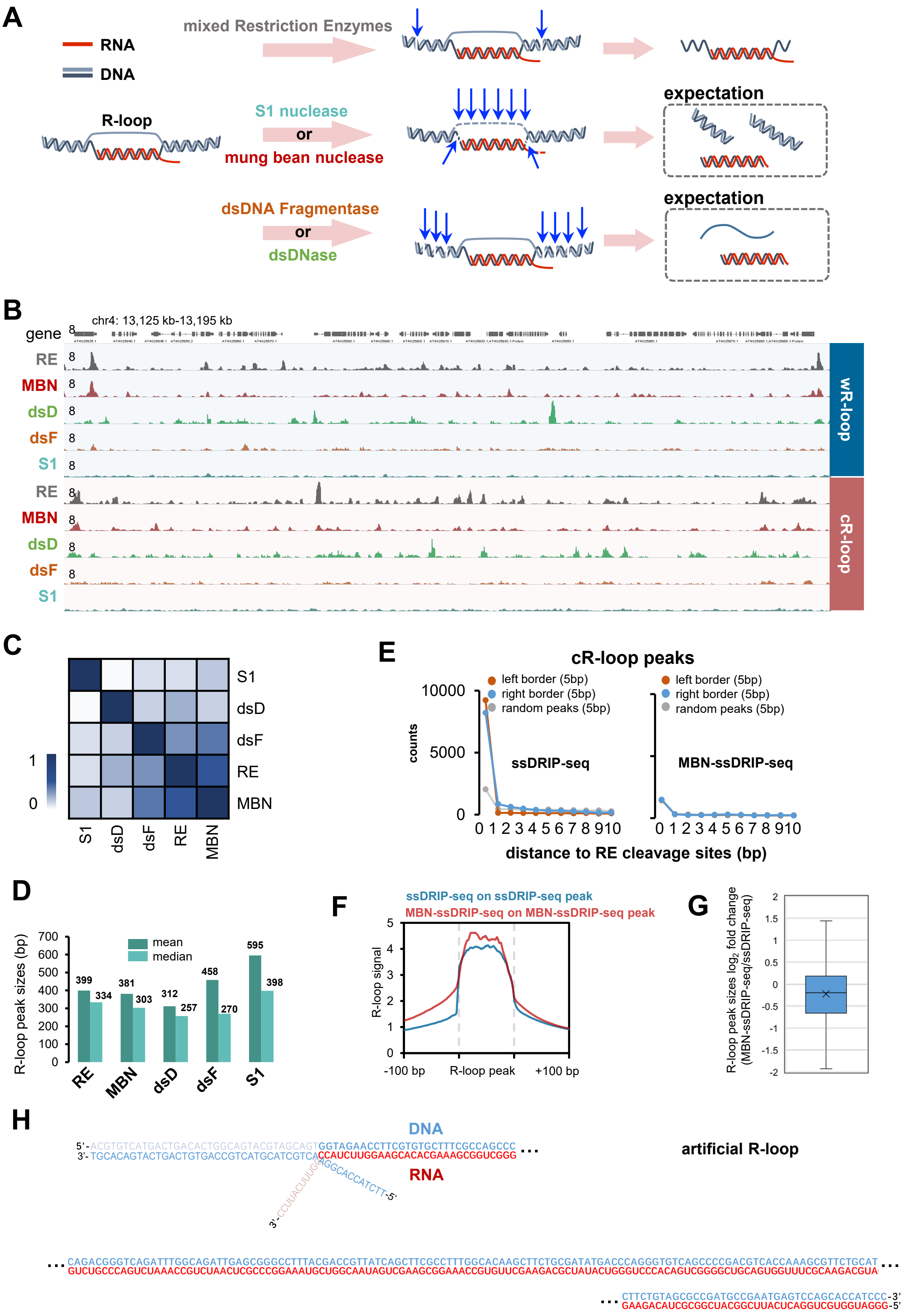
A
RNA
DNA
mixed Restriction Enzymes
R-loop
S1 nuclease
or
mung bean nuclease
expectation
dsDNA Fragmentase
or
dsDNase
expectation
B
chr4: 13,125 kb-13,195 kb
gene
RE
MBN
dsD
dsF
S1
wR-loop
cR-loop
C
S1
dsD
dsF
RE
MBN
E
cR-loop peaks
left border (5bp)
right border (5bp)
random peaks (5bp)
ssDRIP-seq
MBN-ssDRIP-seq
counts
distance to RE cleavage sites (bp)
D
R-loop peak sizes (bp)
mean
median
399 334 381 303 312 257 458 270 595 398
RE MBN dsD dsF S1
F
ssDRIP-seq on ssDRIP-seq peak
MBN-ssDRIP-seq on MBN-ssDRIP-seq peak
R-loop signal
-100 bp
R-loop peak
+100 bp
G
R-loop peak sizes log2 fold change (MBN-ssDRIP-seq/ssDRIP-seq)
H
DNA
RNA
artificial R-loop

Figure S1

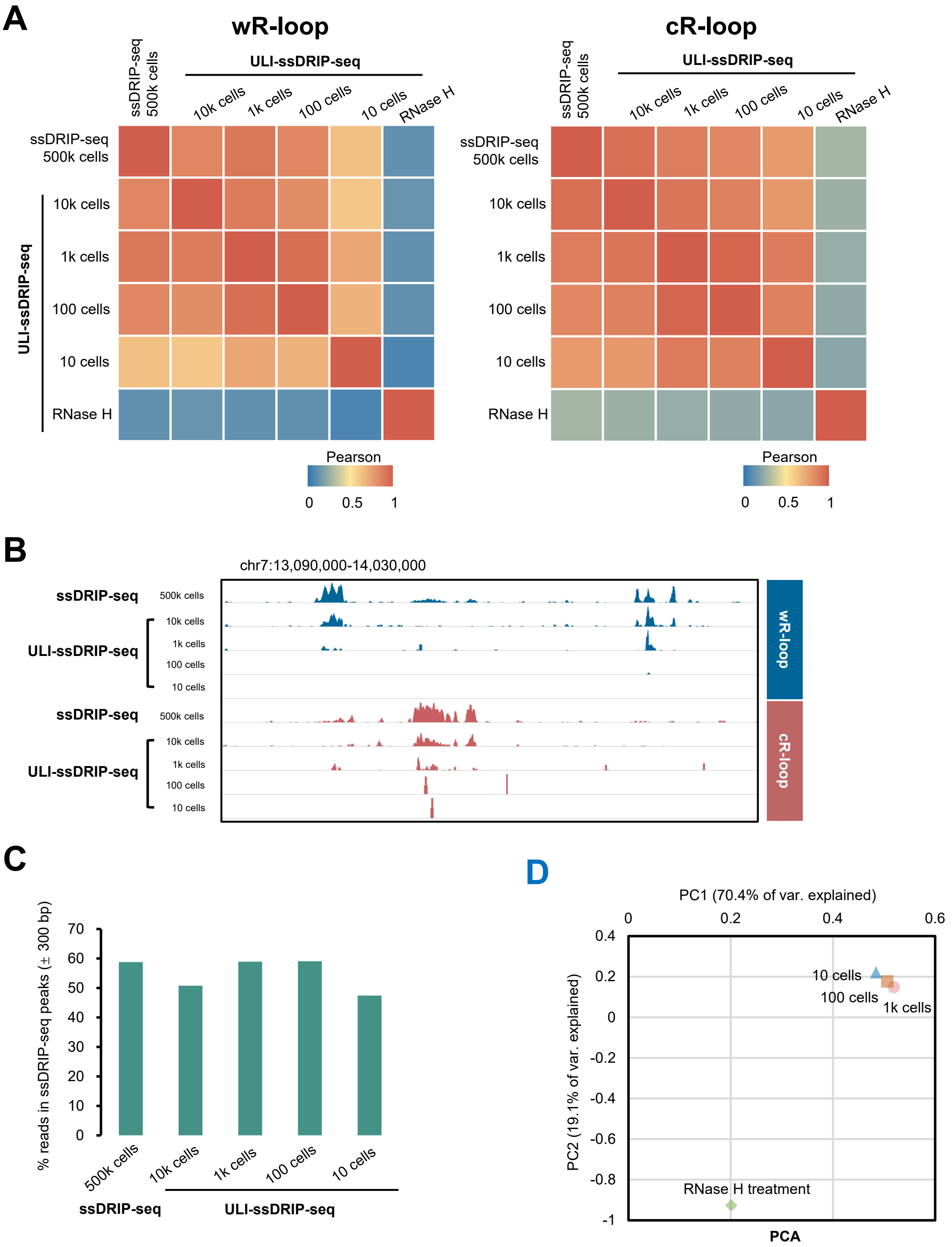
A
wR-loop
cR-loop
ULI-ssDRIP-seq
ssDRIP-seq 500k cells
10k cells
1k cells
100 cells
10 cells
RNase H
Pearson
0
0.5
1
B
chr7:13,090,000-14,030,000
ssDRIP-seq
ULI-ssDRIP-seq
500k cells
wR-loop
cR-loop
C
% reads in ssDRIP-seq peaks (± 300 bp)
70
60
50
40
30
20
10
0
D
PC1 (70.4% of var. explained)
PC2 (19.1% of var. explained)
RNase H treatment
PCA

Figure S2

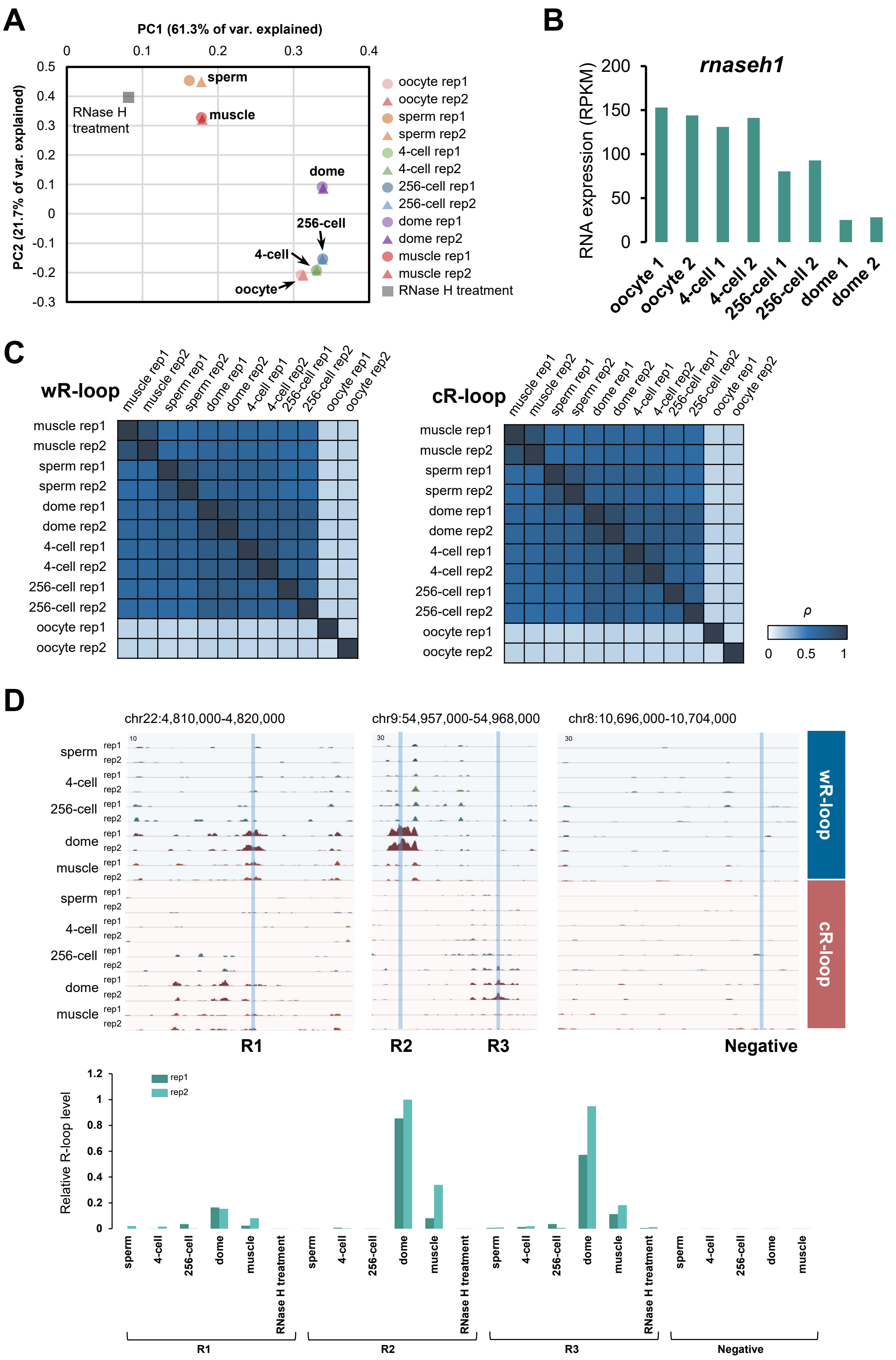

A
PC1 (61.3% of var. explained)
PC2 (21.7% of var. explained)
RNase H treatment
sperm
muscle
dome
256-cell
4-cell
oocyte
oocyte rep1
oocyte rep2
sperm rep1
sperm rep2
4-cell rep1
4-cell rep2
256-cell rep1
256-cell rep2
dome rep1
dome rep2
muscle rep1
muscle rep2
RNase H treatment
B
rnaseh1
RNA expression (RPKM)
oocyte 1
oocyte 2
4-cell 1
4-cell 2
256-cell 1
256-cell 2
dome 1
dome 2
C
wR-loop
cR-loop
muscle rep1
muscle rep2
sperm rep1
sperm rep2
dome rep1
dome rep2
4-cell rep1
4-cell rep2
256-cell rep1
256-cell rep2
oocyte rep1
oocyte rep2
ρ
0
0.5
1
D
chr22:4,810,000-4,820,000
chr9:54,957,000-54,968,000
chr8:10,696,000-10,704,000
wR-loop
cR-loop
R1
R2
R3
Negative
Relative R-loop level
rep1
rep2
RNase H treatment

Figure S3

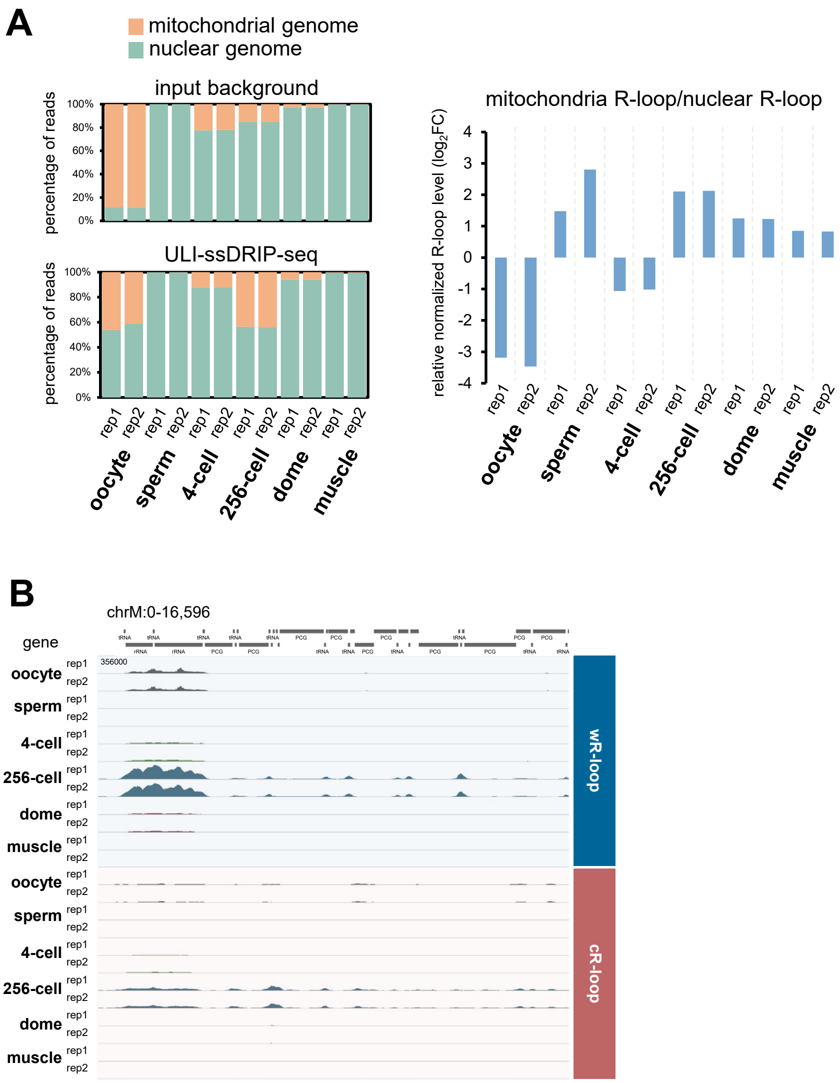
A
mitochondrial genome
nuclear genome
input background
percentage of reads
100%
80%
60%
40%
20%
0%
ULI-ssDRIP-seq
rep1
rep2
oocyte
sperm
4-cell
256-cell
dome
muscle
mitochondria R-loop/nuclear R-loop
relative normalized R-loop level (log2FC)
B
chrM:0-16,596
gene
tRNA
rRNA
PCG
356000
wR-loop
cR-loop

Figure S4

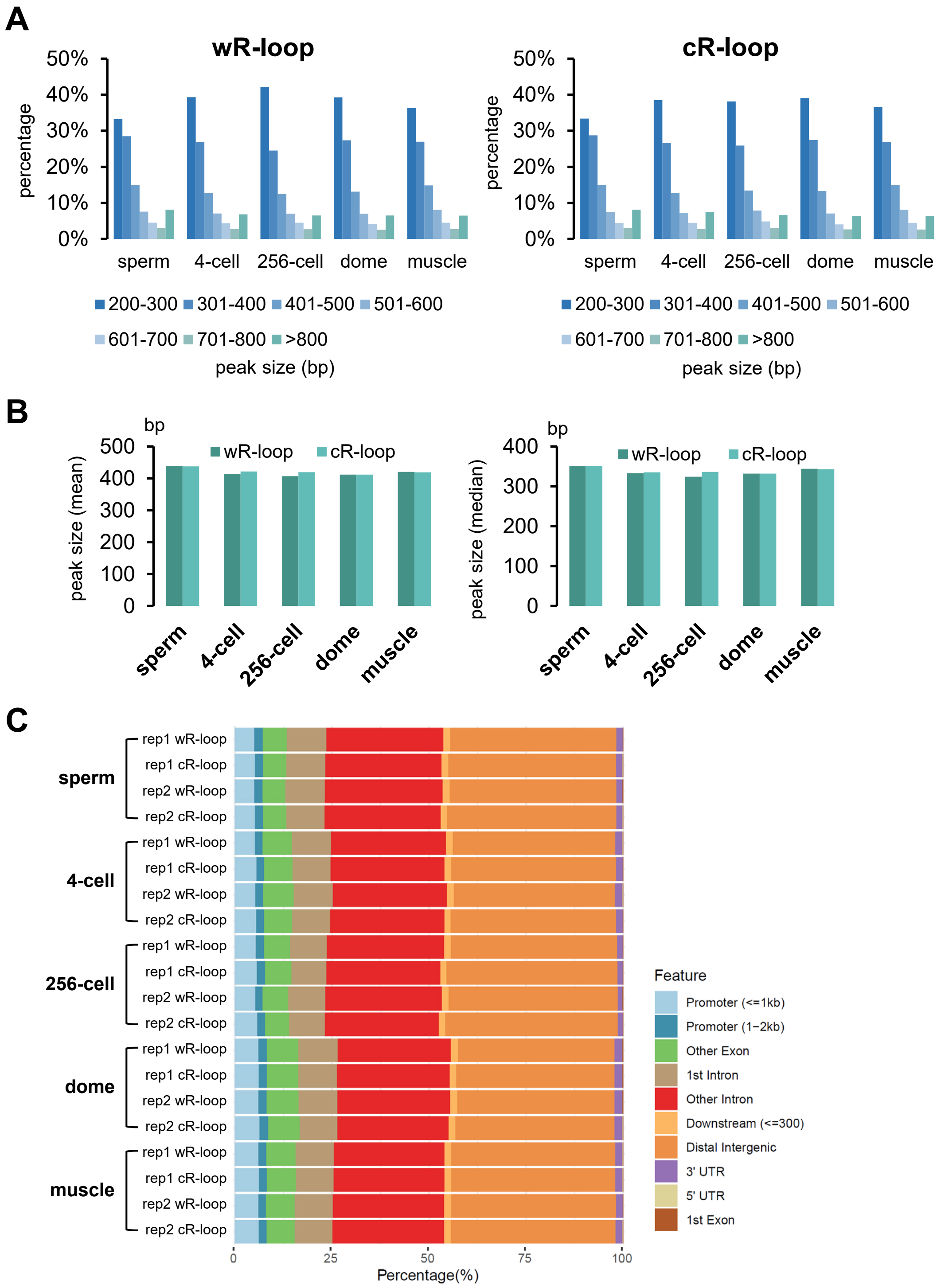
A
wR-loop
cR-loop
percentage
50%
40%
30%
20%
10%
0%
sperm
4-cell
256-cell
dome
muscle
200-300
301-400
401-500
501-600
601-700
701-800
>800
peak size (bp)
B
bp
peak size (mean)
peak size (median)
500
400
300
200
100
0
wR-loop
cR-loop
C
rep1 wR-loop
rep1 cR-loop
rep2 wR-loop
rep2 cR-loop
Feature
Promoter (<=1kb)
Promoter (1–2kb)
Other Exon
1st Intron
Other Intron
Downstream (<=300)
Distal Intergenic
3' UTR
5' UTR
1st Exon
0
25
50
75
100
Percentage(%)

Figure S5

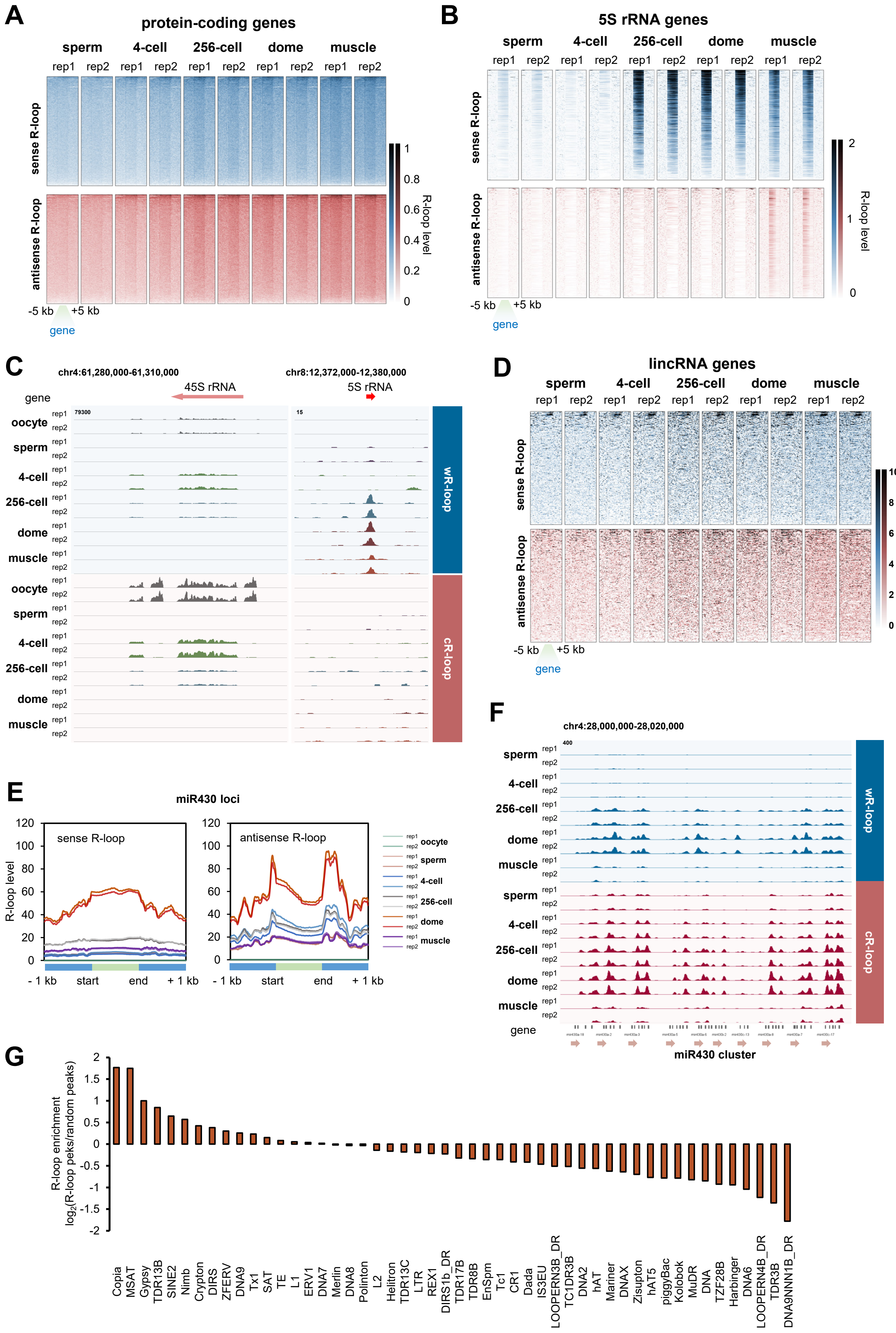
A
protein-coding genes
sperm
4-cell
256-cell
dome
muscle
rep1
rep2
sense R-loop
antisense R-loop
R-loop level
-5 kb
+5 kb
gene
B
5S rRNA genes
C
chr4:61,280,000-61,310,000
chr8:12,372,000-12,380,000
45S rRNA
5S rRNA
oocyte
wR-loop
cR-loop
D
lincRNA genes
E
miR430 loci
sense R-loop
antisense R-loop
R-loop level
- 1 kb
start
end
+ 1 kb
F
chr4:28,000,000-28,020,000
miR430 cluster
G
R-loop enrichment
log2(R-loop peks/random peaks)
Copia
MSAT
Gypsy
TDR13B
SINE2
Nimb
Crypton
DIRS
ZFERV
DNA9
Tx1
SAT
TE
L1
ERV1
DNA7
Merlin
DNA8
Polinton
L2
Helitron
TDR13C
LTR
REX1
DIRS1b_DR
TDR17B
TDR8B
EnSpm
Tc1
CR1
Dada
IS3EU
LOOPERN3B_DR
TC1DR3B
DNA2
hAT
Mariner
DNAX
Zisupton
hAT5
piggyBac
Kolobok
MuDR
DNA
TZF28B
Harbinger
DNA6
LOOPERN4B_DR
TDR3B
DNA9NNN1B_DR

Figure S6

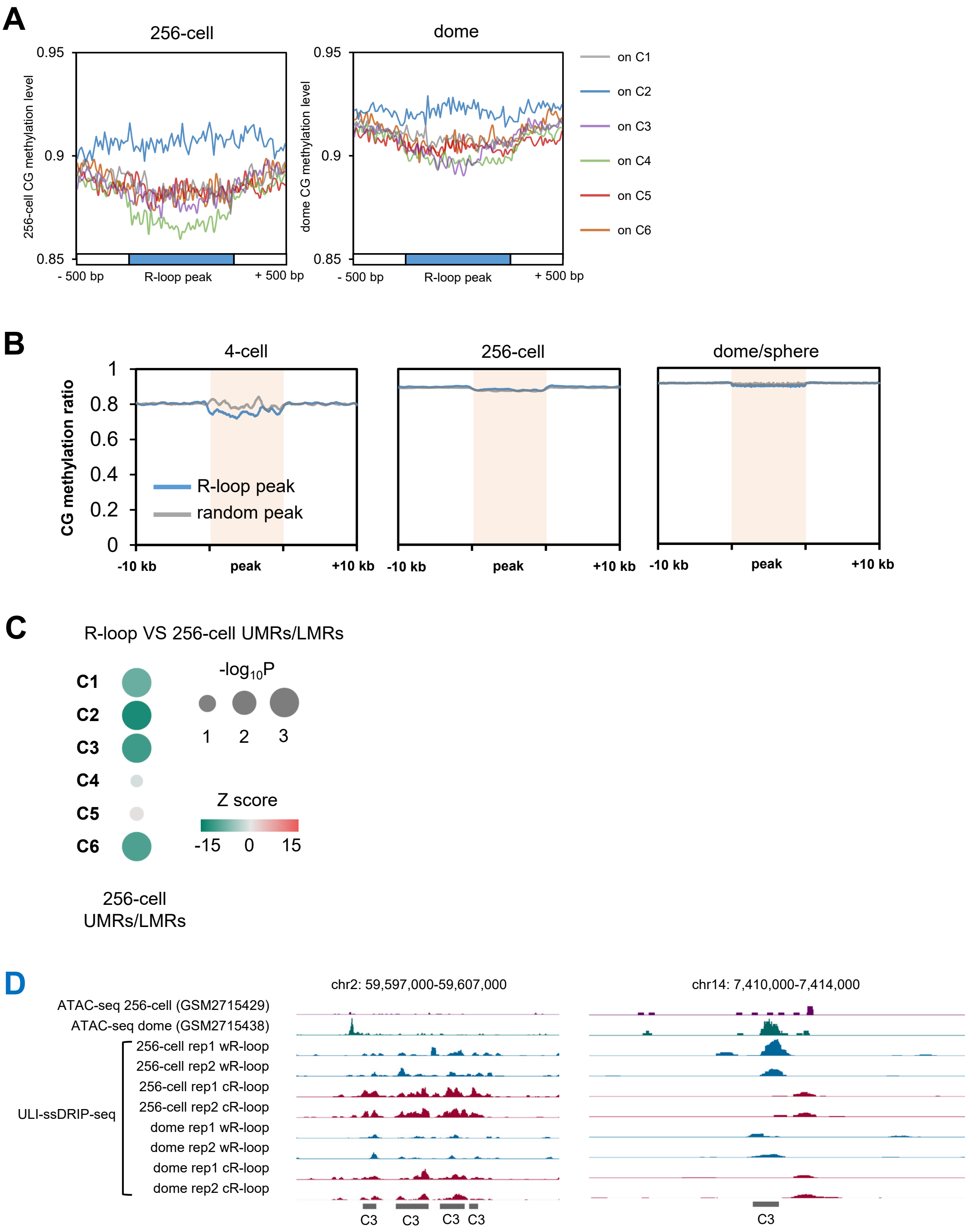

A
256-cell
dome
0.95
0.9
0.85
256-cell CG methylation level
dome CG methylation level
- 500 bp
R-loop peak
+ 500 bp
on C1
on C2
on C3
on C4
on C5
on C6
B
4-cell
256-cell
dome/sphere
CG methylation ratio
1
0.8
0.6
0.4
0.2
0
R-loop peak
random peak
-10 kb
peak
+10 kb
C
R-loop VS 256-cell UMRs/LMRs
C1
C2
C3
C4
C5
C6
-log10P
1
2
3
Z score
-15
0
15
256-cell UMRs/LMRs
D
chr2: 59,597,000-59,607,000
chr14: 7,410,000-7,414,000
ATAC-seq 256-cell (GSM2715429)
ATAC-seq dome (GSM2715438)
ULI-ssDRIP-seq
256-cell rep1 wR-loop
256-cell rep2 wR-loop
256-cell rep1 cR-loop
256-cell rep2 cR-loop
dome rep1 wR-loop
dome rep2 wR-loop
dome rep1 cR-loop
dome rep2 cR-loop
C3
C3
C3
C3
C3

Figure S7

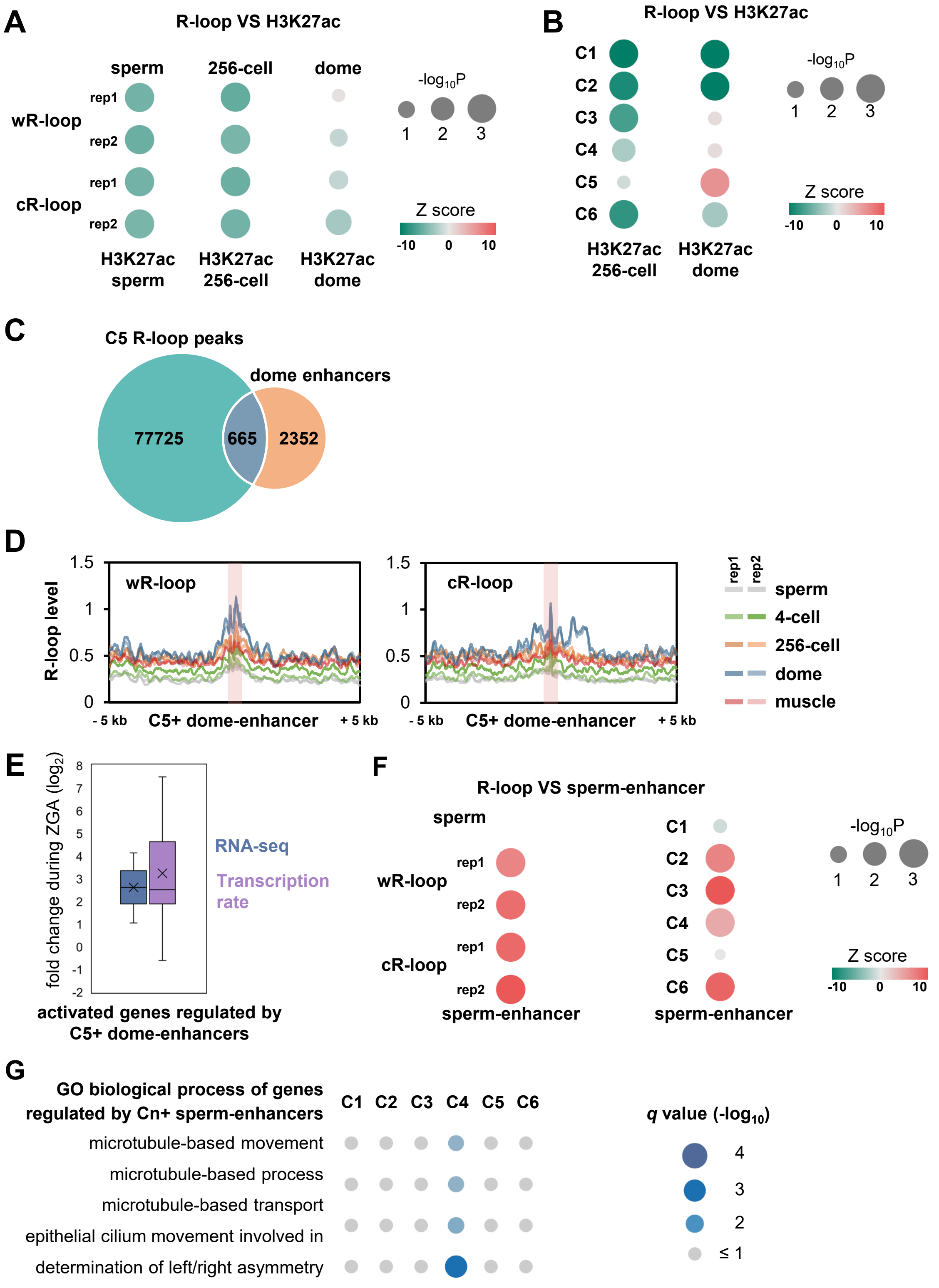

A
R-loop VS H3K27ac
sperm
256-cell
dome
wR-loop
cR-loop
rep1
rep2
rep1
rep2
H3K27ac sperm
H3K27ac 256-cell
H3K27ac dome
-log10P
1 2 3
Z score
-10 0 10
B
R-loop VS H3K27ac
C1
C2
C3
C4
C5
C6
H3K27ac 256-cell
H3K27ac dome
C
C5 R-loop peaks
dome enhancers
77725
665
2352
D
R-loop level
wR-loop
cR-loop
1.5
1
0.5
0
- 5 kb
C5+ dome-enhancer
+ 5 kb
rep1
rep2
sperm
4-cell
256-cell
dome
muscle
E
fold change during ZGA (log2)
RNA-seq
Transcription rate
activated genes regulated by C5+ dome-enhancers
F
R-loop VS sperm-enhancer
sperm
sperm-enhancer
G
GO biological process of genes regulated by Cn+ sperm-enhancers
C1 C2 C3 C4 C5 C6
microtubule-based movement
microtubule-based process
microtubule-based transport
epithelial cilium movement involved in determination of left/right asymmetry
q value (-log10)
4
3
2
≤ 1

Figure S8

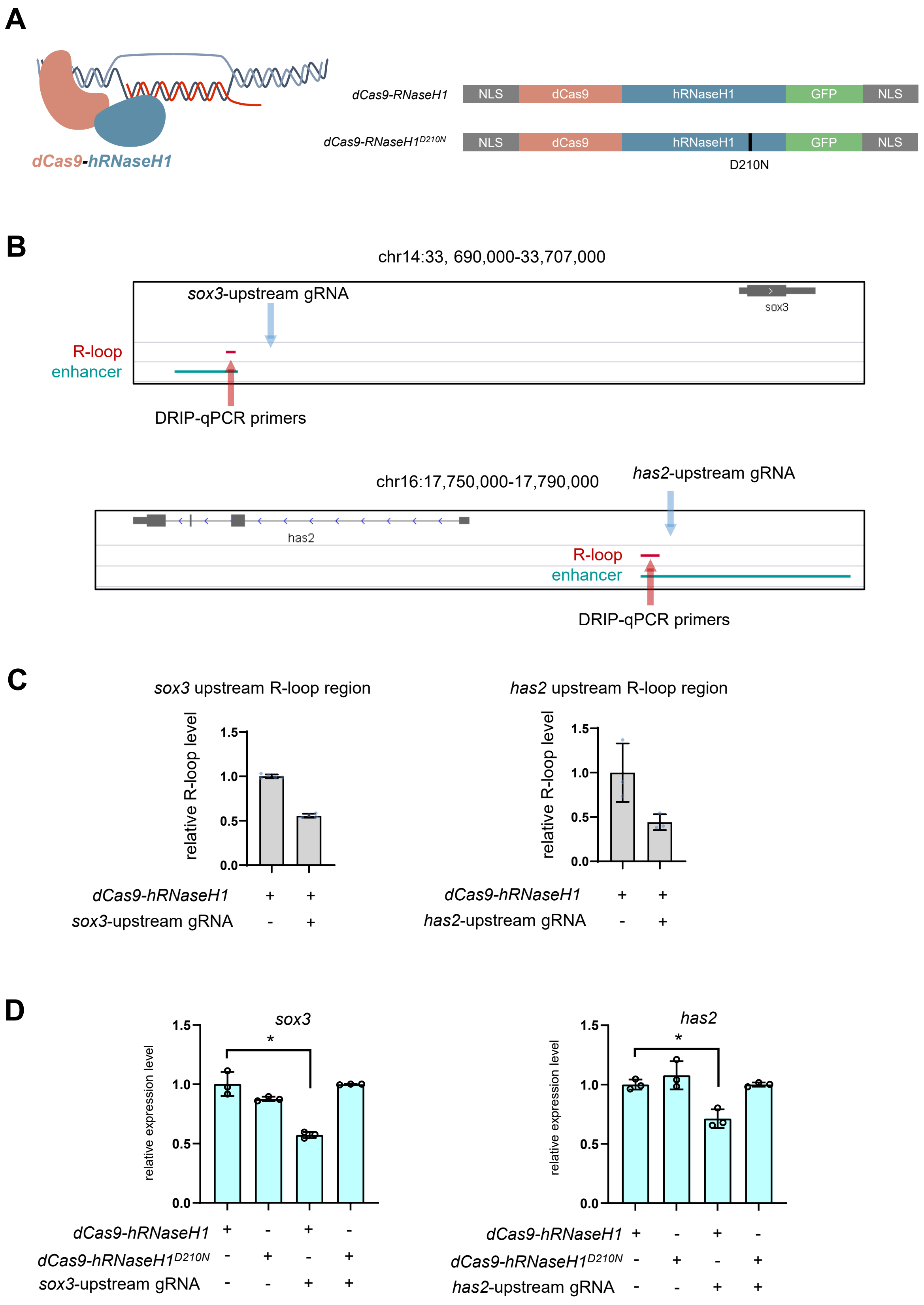
A
dCas9-hRNaseH1
dCas9-RNaseH1
dCas9-RNaseH1D210N
NLS
dCas9
hRNaseH1
GFP
NLS
D210N
B
chr14:33, 690,000-33,707,000
sox3-upstream gRNA
sox3
R-loop
enhancer
DRIP-qPCR primers
chr16:17,750,000-17,790,000
has2-upstream gRNA
has2
R-loop
enhancer
DRIP-qPCR primers
C
sox3 upstream R-loop region
has2 upstream R-loop region
relative R-loop level
1.5
1.0
0.5
0.0
dCas9-hRNaseH1 + +
sox3-upstream gRNA - +
has2-upstream gRNA - +
D
sox3
has2
*
relative expression level
dCas9-hRNaseH1 + - + -
dCas9-hRNaseH1D210N - + - +
sox3-upstream gRNA - - + +
has2-upstream gRNA - - + +

Figure S9

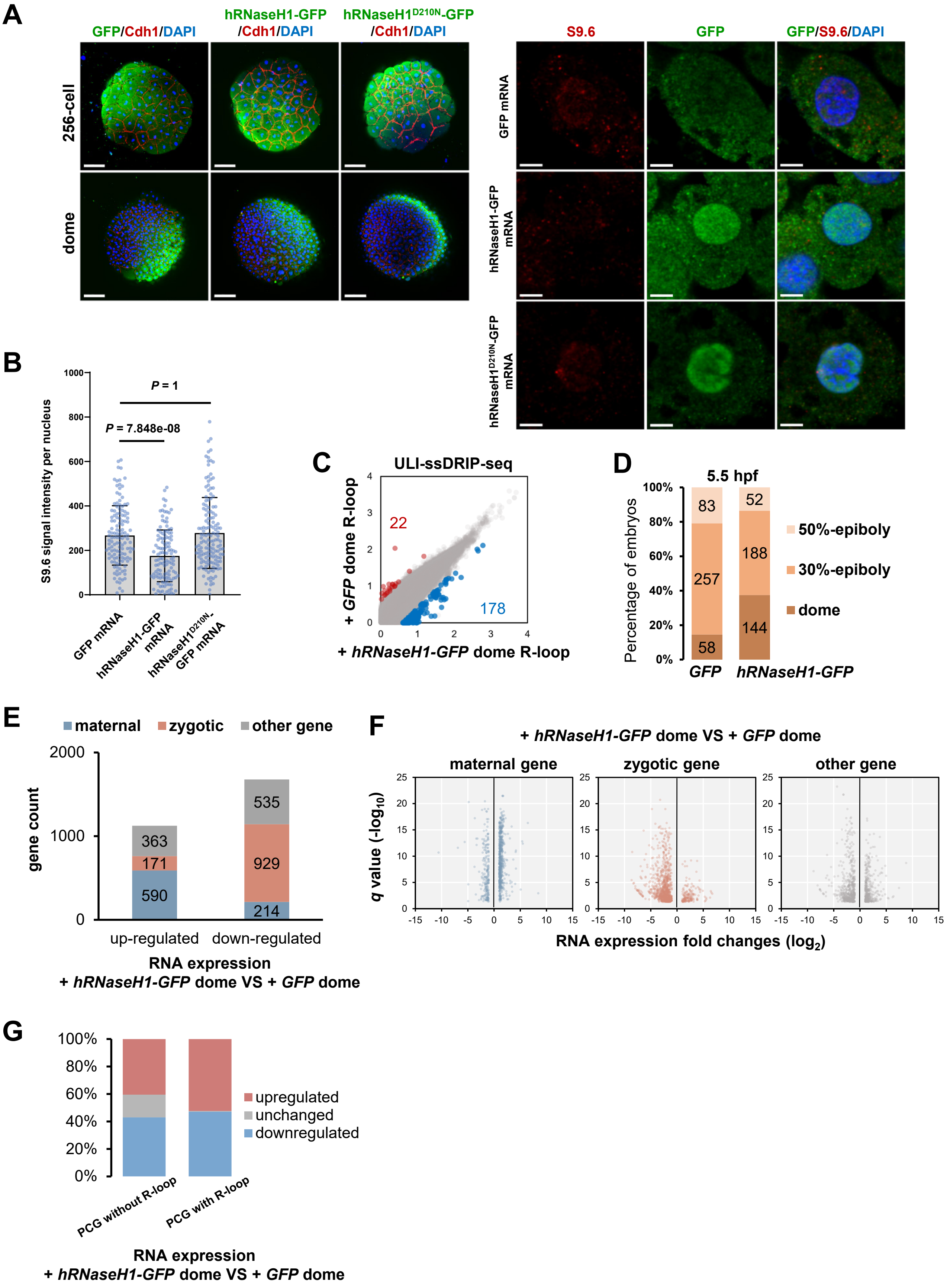

A
GFP/Cdh1/DAPI
hRNaseH1-GFP /Cdh1/DAPI
hRNaseH1D210N-GFP /Cdh1/DAPI
256-cell
dome
S9.6
GFP
GFP/S9.6/DAPI
GFP mRNA
hRNaseH1-GFP mRNA
hRNaseH1D210N-GFP mRNA
B
S9.6 signal intensity per nucleus
P = 1
P = 7.848e-08
GFP mRNA
hRNaseH1-GFP mRNA
hRNaseH1D210N-GFP mRNA
C
ULI-ssDRIP-seq
+ GFP dome R-loop
+ hRNaseH1-GFP dome R-loop
22
178
D
5.5 hpf
Percentage of embryos
83
257
58
52
188
144
50%-epiboly
30%-epiboly
dome
GFP
hRNaseH1-GFP
E
maternal
zygotic
other gene
gene count
363
171
590
535
929
214
up-regulated
down-regulated
RNA expression
+ hRNaseH1-GFP dome VS + GFP dome
F
+ hRNaseH1-GFP dome VS + GFP dome
maternal gene
zygotic gene
other gene
q value (-log10)
RNA expression fold changes (log2)
G
upregulated
unchanged
downregulated
PCG without R-loop
PCG with R-loop
RNA expression
+ hRNaseH1-GFP dome VS + GFP dome

Figure S10

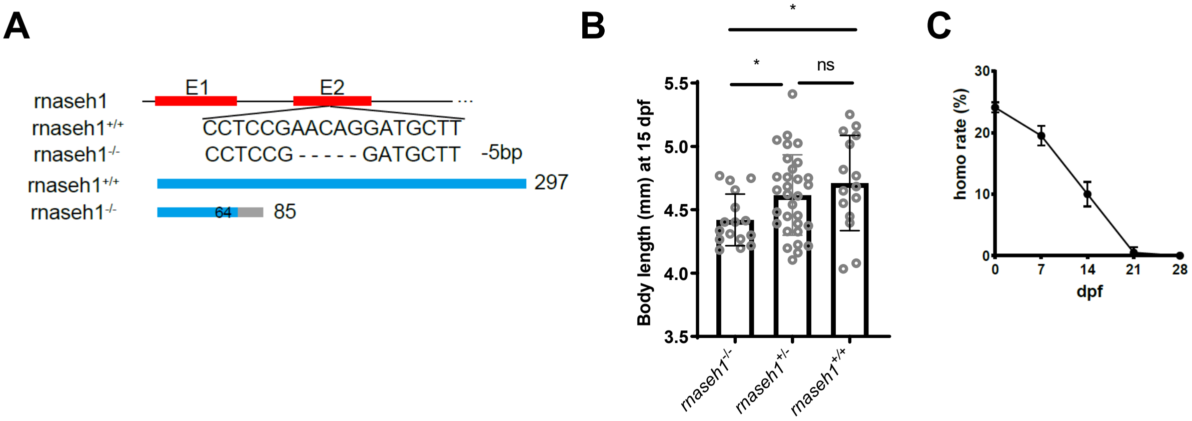
A
rnaseh1
E1
E2
rnaseh1+/+
CCTCCGAACAGGATGCTT
rnaseh1-/-
CCTCCG - - - - - GATGCTT
-5bp
rnaseh1+/+
297
rnaseh1-/-
64
85
B
*
*
ns
Body length (mm) at 15 dpf
5.5
5.0
4.5
4.0
3.5
rnaseh1-/-
rnaseh1+/-
rnaseh1+/+
C
homo rate (%)
30
20
10
0
0
7
14
21
28
dpf

Figure S11

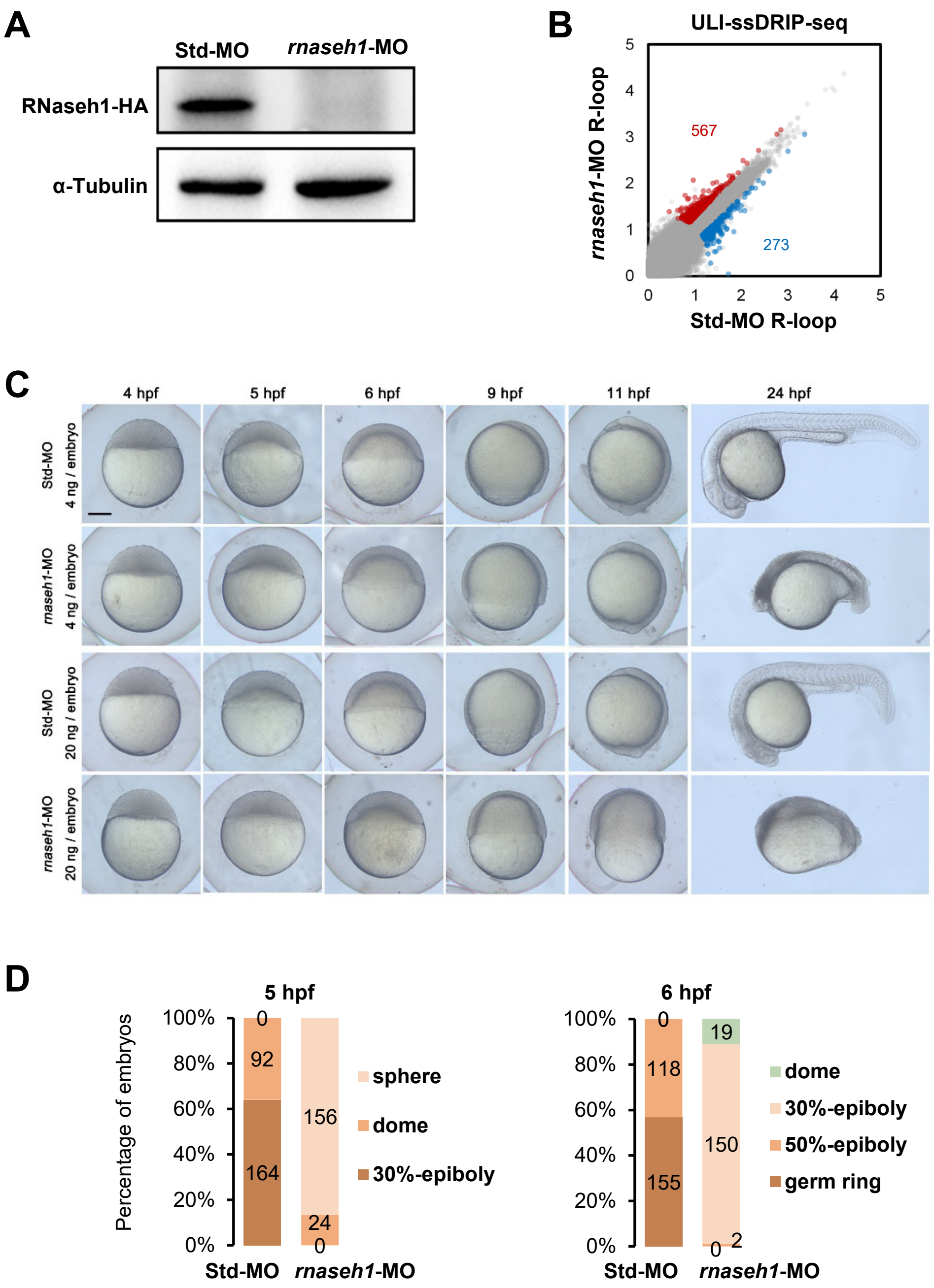
A
Std-MO
rnaseh1-MO
RNaseh1-HA
α-Tubulin
B
ULI-ssDRIP-seq
rnaseh1-MO R-loop
Std-MO R-loop
567
273
C
4 hpf
5 hpf
6 hpf
9 hpf
11 hpf
24 hpf
Std-MO 4 ng / embryo
rnaseh1-MO 4 ng / embryo
Std-MO 20 ng / embryo
rnaseh1-MO 20 ng / embryo
D
Percentage of embryos
5 hpf
6 hpf
sphere
dome
30%-epiboly
50%-epiboly
germ ring
Std-MO
rnaseh1-MO

Figure S12

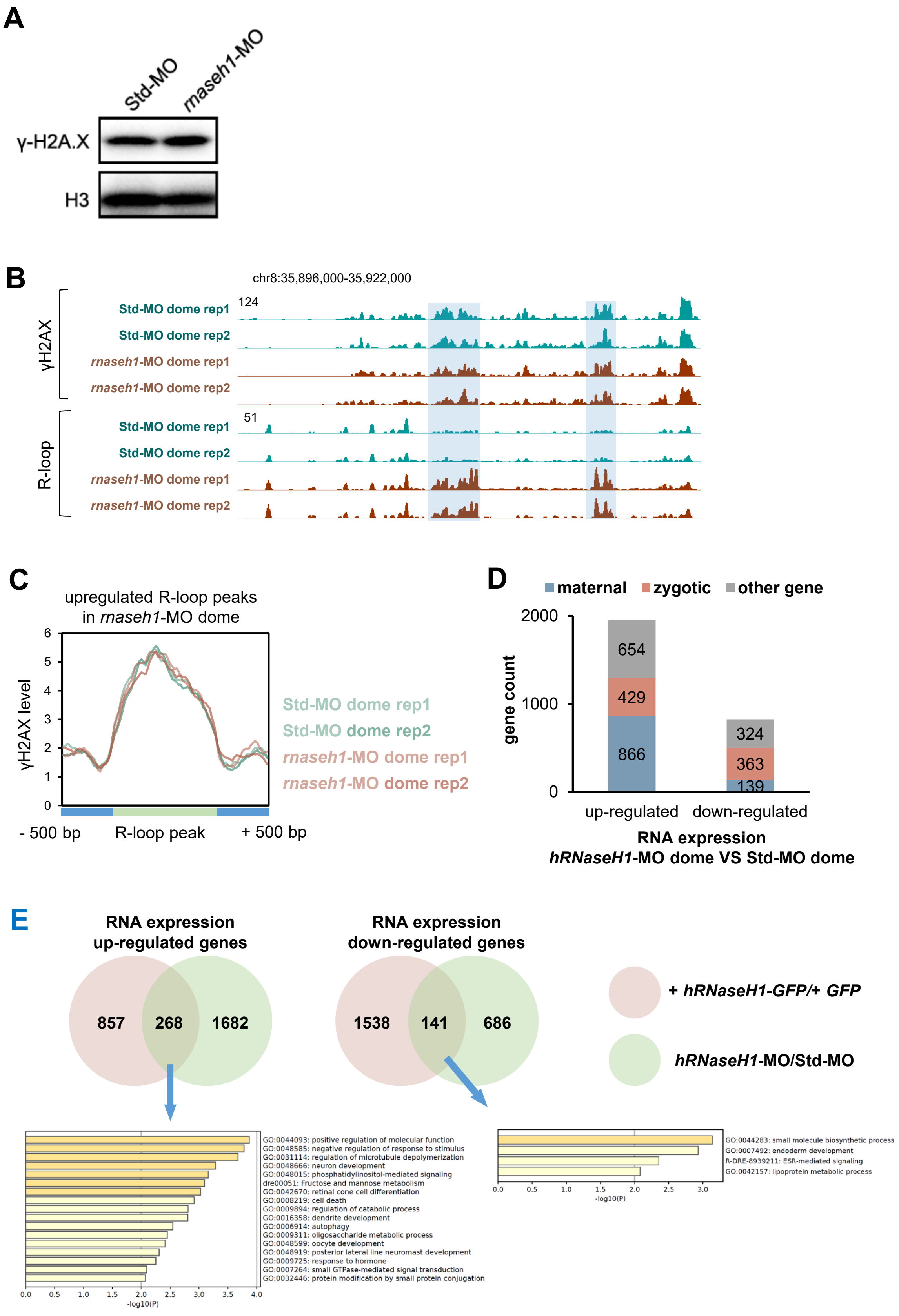
A
Std-MO
rnaseh1-MO
γ-H2A.X
H3
B
chr8:35,896,000-35,922,000
γH2AX
124
Std-MO dome rep1
Std-MO dome rep2
rnaseh1-MO dome rep1
rnaseh1-MO dome rep2
R-loop
51
Std-MO dome rep1
Std-MO dome rep2
rnaseh1-MO dome rep1
rnaseh1-MO dome rep2
C
upregulated R-loop peaks
in rnaseh1-MO dome
γH2AX level
6
5
4
3
2
1
0
- 500 bp
R-loop peak
+ 500 bp
Std-MO dome rep1
Std-MO dome rep2
rnaseh1-MO dome rep1
rnaseh1-MO dome rep2
D
maternal
zygotic
other gene
gene count
2000
1000
0
654
429
866
324
363
139
up-regulated
down-regulated
RNA expression
hRNaseH1-MO dome VS Std-MO dome
E
RNA expression
up-regulated genes
857
268
1682
RNA expression
down-regulated genes
1538
141
686
+ hRNaseH1-GFP/+ GFP
hRNaseH1-MO/Std-MO
GO:0044093: positive regulation of molecular function
GO:0048585: negative regulation of response to stimulus
GO:0031114: regulation of microtubule depolymerization
GO:0048666: neuron development
GO:0048015: phosphatidylinositol-mediated signaling
dre00051: Fructose and mannose metabolism
GO:0042670: retinal cone cell differentiation
GO:0008219: cell death
GO:0009894: regulation of catabolic process
GO:0016358: dendrite development
GO:0006914: autophagy
GO:0009311: oligosaccharide metabolic process
GO:0048599: oocyte development
GO:0048919: posterior lateral line neuromast development
GO:0009725: response to hormone
GO:0007264: small GTPase-mediated signal transduction
GO:0032446: protein modification by small protein conjugation
0.0
0.5
1.0
1.5
2.0
2.5
3.0
3.5
4.0
-log10(P)
GO:0044283: small molecule biosynthetic process
GO:0007492: endoderm development
R-DRE-8939211: ESR-mediated signaling
GO:0042157: lipoprotein metabolic process
0.0
0.5
1.0
1.5
2.0
2.5
3.0
-log10(P)

Figure S13

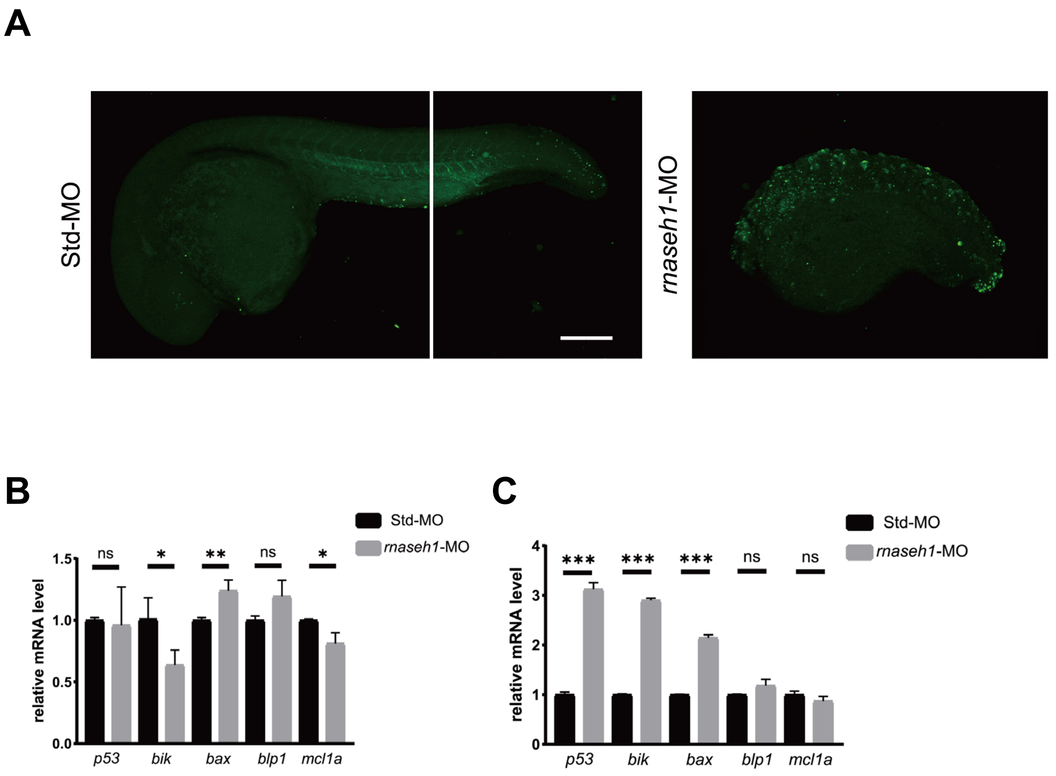
A
Std-MO
rnaseh1-MO
B
Std-MO
rnaseh1-MO
relative mRNA level
1.5
1.0
0.5
0.0
ns
*
**
ns
*
p53
bik
bax
blp1
mcl1a
C
Std-MO
rnaseh1-MO
relative mRNA level
4
3
2
1
0
***
***
***
ns
ns
p53
bik
bax
blp1
mcl1a

Figure S14
